# Multi-Constraint Three-Dimensional Bin Packing Optimization for Mixed Vehicle Types: A Heuristic Approach

**DOI:** 10.3390/e28080905

**Published:** 2026-08-12

**Authors:** Yiting Hao, Dongqing Cao, Wenhao Gui

**Affiliations:** School of Mathematics and Statistics, Beijing Jiaotong University, Beijing 100044, China; 23271033@bjtu.edu.cn (Y.H.); 23271028@bjtu.edu.cn (D.C.)

**Keywords:** three-dimensional bin packing, mixed vehicle scheduling, heuristic algorithm, multi-constraint optimization, sensitivity analysis, NSGA-II

## Abstract

Aiming at the multi-constraint three-dimensional bin packing problem for mixed vehicle types in urban logistics, where traditional exact algorithms are limited by NP-hard computational complexity and practical engineering constraints, this study proposes a progressive optimization framework for vehicle loading and fleet allocation optimization. First, a heuristic loading algorithm based on the extreme point method and greedy strategy is developed to maximize single-vehicle loading efficiency by balancing space and weight utilization. Second, an NSGA-II based evolutionary framework with sequential encoding is constructed to minimize fleet size while improving loading balance for single-vehicle-type optimization. Third, a three-stage hybrid algorithm integrating greedy packing, enumerative search, and tail vehicle replacement is designed to optimize mixed-vehicle fleet composition and minimize total transportation cost. Experimental results demonstrate that the proposed heuristic achieves high composite loading performance across vehicle types, and the evolutionary framework significantly reduces fleet size compared with theoretical lower bounds. Under mixed-fleet optimization, the model identifies cost-effective vehicle configurations that outperform single-type dispatching strategies. Sensitivity analysis reveals that cargo composition, particularly the number of fragile items, is the most critical factor affecting system performance, while validation on 16 vehicle types confirms the robustness and practical generalizability of the method. This study verifies the effectiveness and stability of heuristic-evolutionary hybrid optimization methods, providing a reliable decision-making reference for logistics enterprises in vehicle selection, cargo allocation, and transportation planning.

## 1. Introduction

As global trade and e-commerce continue to expand, logistics efficiency has become a critical determinant of economic competitiveness worldwide. Globally, logistics costs represent approximately 10–15% of GDP in developed economies and 15–20% in developing economies, accounting for an estimated 2.5–3.0 trillion USD annually worldwide [1]. In the United States, the logistics sector accounted for approximately 9.1% of GDP in 2022, with total business logistics costs reaching 2.3 trillion USD, driven largely by freight transportation and warehousing inefficiencies [2]. In the European Union, logistics costs represent around 10-12% of GDP, with urban last-mile delivery inefficiencies accounting for a substantial portion [3]. In China, according to the China Logistics Yearbook 2024, logistics costs accounted for 14.4% of GDP in 2023, with transportation and loading inefficiencies contributing an estimated 2–3% of this total [4]. These figures demonstrate that logistics inefficiency is a global challenge, affecting both developed and developing economies, and motivating research efforts across different regions.

Reducing these logistics costs depends critically on loading optimization [5,6]. The core challenge is to arrange cargo within limited space under multiple constraints—dimensions, weight, load-bearing capacity, stacking rules, and orientation restrictions—to maximize both space and weight utilization [7,8]. In real-world logistics, these constraints take various forms. Fragile items require single-layer placement with no stacking above [9,10]; directional items require fixed orientations with constrained centroid projection during stacking [11]; and standard carton items are rotatable but must satisfy load-bearing limits [12,13]. These engineering constraints dramatically increase problem complexity, making exact algorithms impractical for large instances [14,15].

This problem is known as the Three-Dimensional Bin Packing Problem (3D-BPP), a classic NP-hard combinatorial optimization problem [16,17]. Research on 3D-BPP dates back to systematic modeling [16] and early computational work. The basic formulation packs rectangular boxes of different sizes into the minimum number of containers.

Heuristic and evolutionary methods have been widely applied to 3D-BPP. Ref. [5] systematically reviewed practical constraints, highlighting stacking stability and load limits as bridges between theory and practice. The extreme point method [18] provides an efficient framework for placement decisions in three-dimensional spaces. Various metaheuristic approaches have been explored for related packing problems. Genetic algorithms are among the most widely used methods because of their flexibility in encoding packing sequences and searching large solution spaces [12,13]. Tabu search has also demonstrated competitive performance by effectively exploring neighborhood structures and avoiding premature convergence [18,19]. In addition, population-based and trajectory-based metaheuristics, including ant colony optimization, particle swarm optimization, and simulated annealing, have been investigated for solving packing and combinatorial optimization problems [20,21,22]. More recently, hybrid metaheuristics that integrate global search with problem-specific heuristics have attracted increasing attention because of their improved balance between exploration and exploitation in large-scale optimization problems [23,24]. For multi-objective logistics optimization, NSGA-II has become one of the most widely adopted evolutionary algorithms because of its efficient non-dominated sorting and crowding-distance mechanisms for maintaining solution diversity [25,26,27,28]. In this study, NSGA-II is adopted to optimize two conflicting objectives, namely minimizing fleet size and maximizing loading balance, without requiring scalarization of the objective functions.

However, three important gaps persist. The first concerns fleet homogeneity: most studies assume a single vehicle type, leaving mixed-vehicle collaborative scheduling largely unexplored [29,30]. The second relates to objective integration: space utilization, weight utilization, transportation cost, and cargo heterogeneity are rarely addressed within a unified multi-objective framework [10,31]. The third involves decision-making: few models translate algorithmic outputs into actionable managerial insights [32,33].

Closing these gaps has both theoretical and practical value. This paper develops optimization models and algorithms for mixed vehicle types under realistic constraints, including standard, fragile, and directional cargo. The proposed framework is designed to handle practical engineering requirements, although some real-world complexities, such as irregularly shaped cargo and non-ideal compartment geometries, are simplified in the current implementation to maintain computational tractability. These simplifications are discussed in Section 4.7, where their implications are addressed and possible extensions are suggested. Within this defined scope, the proposed approach offers a systematic and practical solution for urban logistics loading and fleet allocation [6,32,34].

The novelty of the proposed framework lies not in the invention of entirely new optimization primitives, but in the systematic integration and adaptation of existing techniques to address a class of problems that has not been adequately covered in the literature. While extreme-point methods and NSGA-II have been applied to 3D bin packing separately, and mixed-fleet optimization has been studied in routing contexts, the combination of these components into a unified progressive framework for multi-constraint, mixed-vehicle-type loading has not been explored. The key contribution is the architecture itself: the three-stage decomposition (single-vehicle loading → single-vehicle-type fleet optimization → mixed-vehicle-type cost optimization) allows each stage to use the most suitable technique, while the output of each stage provides a feasible and well-structured starting point for the next. This design is motivated by the observation that real-world logistics decisions are naturally hierarchical: loading plans determine vehicle requirements, which in turn determine fleet composition. By aligning the optimization stages with this practical decision hierarchy, the framework achieves both computational efficiency and solution quality without requiring problem-specific parameter tuning.

The main contributions are fourfold.

(1) A heuristic loading algorithm. Based on the extreme point method and greedy strategy, it prioritizes cargo by volume- and density-weighted ranking to optimize space and weight simultaneously. Unlike existing extreme point methods that focus only on volume, our approach explicitly balances both dimensions through a composite ranking criterion that accounts for both spatial and weight efficiency in a single pass.

(2) An NSGA-II based evolutionary framework. With a sequential encoding scheme, it minimizes fleet size while maximizing loading balance. Unlike typical applications of NSGA-II that separate optimization from feasibility evaluation, our framework embeds constraint-aware packing simulation directly into fitness evaluation, ensuring that all Pareto-optimal solutions are physically feasible by construction.

(3) A mixed-fleet collaborative strategy. The “large vehicle first, small vehicle for residual” heuristic, combined with a three-stage hybrid solver (baseline construction, enumerative search, tail vehicle replacement), targets minimum transportation cost. This strategy addresses the mixed-fleet optimization problem without enumerating all possible fleet compositions, making it computationally practical for real-world fleet sizes.

(4) Systematic validation. Sensitivity analysis on six key parameters and validation on 16 vehicle types confirm the model’s generalizability.

The remainder of this paper proceeds as follows. Section 2 develops the mathematical model and solution algorithms. Section 3 presents experimental findings across three problem settings. Section 4 interprets the results and draws practical implications. Section 5 summarizes the study and outlines future directions.

## 2. Materials and Methods

### 2.1. Problem Description and Assumptions

The multi-constraint mixed-vehicle-type three-dimensional bin packing problem studied in this paper can be described as follows: given *n* types of heterogeneous cargo and *m* optional vehicle types, under the constraints of vehicle volume, rated load, cargo orientation, stacking stability, and special handling requirements, determine the optimal vehicle type mix, number of vehicles, and loading plan for each vehicle to optimize space utilization, weight utilization, fleet size, or transportation cost.

Solving this problem has direct economic implications. A 1% improvement in loading efficiency can reduce fleet size by up to 8% in our experiments, translating to annual savings of hundreds of thousands of CNY for medium-sized logistics providers. The trade-off between vehicle types further affects cost structure, making optimal fleet composition a strategic decision.

To establish a solvable mathematical model, the following assumptions are made:

(1) All cargo items are rigid rectangular boxes with fixed and known dimensions and weights.
(2) The vehicle cargo compartment is an ideal regular space; internal protrusions and fixtures are neglected.
(3) Cargo items are placed only in orthogonal orientations without tilting or overlapping.
(4) A fixed safety gap is reserved at the top of the compartment, following GB/T 29912-2024 [35] and GB 1589-2016 [36].
(5) The supporting surface is uniformly loaded. Fragile items require full bottom support and prohibit stacking above. Directional items must satisfy centroid projection constraints [9,10].
(6) Vehicle parameters and unit transportation costs are derived from the China Logistics Yearbook 2024 [4].
(7) All cargo must be loaded in a single shipment; splitting or omission is not permitted.

### 2.2. Comparison with Existing Approaches

To clarify the methodological advances of the proposed framework, Table 1 summarizes the key differences between our approach and representative existing methods in terms of problem scope, optimization strategy, and constraint handling.

### 2.3. Data Sources

Vehicle parameters follow the specifications in GB/T 29912-2024 [35] and GB 1589-2016 [36], covering light-duty vans (gross vehicle weight ≤4.5 t) and medium-duty vans (4.5 t–12 t). Cargo dimensions, weights, and packaging constraints are derived from documented cases in the literature [11,37]. Loading stability and fragility constraints follow the formulations [9,10]. Fleet composition parameters, including vehicle acquisition costs, operating costs, and load limits, are calibrated using industry-wide statistics from the China Logistics Yearbook 2024 [4].

All experiments run on an Intel Xeon 32-core CPU with 128 GB RAM, Ubuntu 22.04 LTS, and Python 3.10. The algorithms were implemented in Python 3.10, and all experiments were conducted under a reproducible computational environment. All enterprise data have been anonymized to protect confidentiality. The SKU statistics represent typical cargo profiles in urban distribution, with dimensions, weights, and quantities reflecting real-world operations.

### 2.4. Notation

The main notation and parameters used throughout this paper are summarized in Table 2. These symbols are consistently applied in the mathematical formulations presented in the subsequent sections.

**Figure 1:**
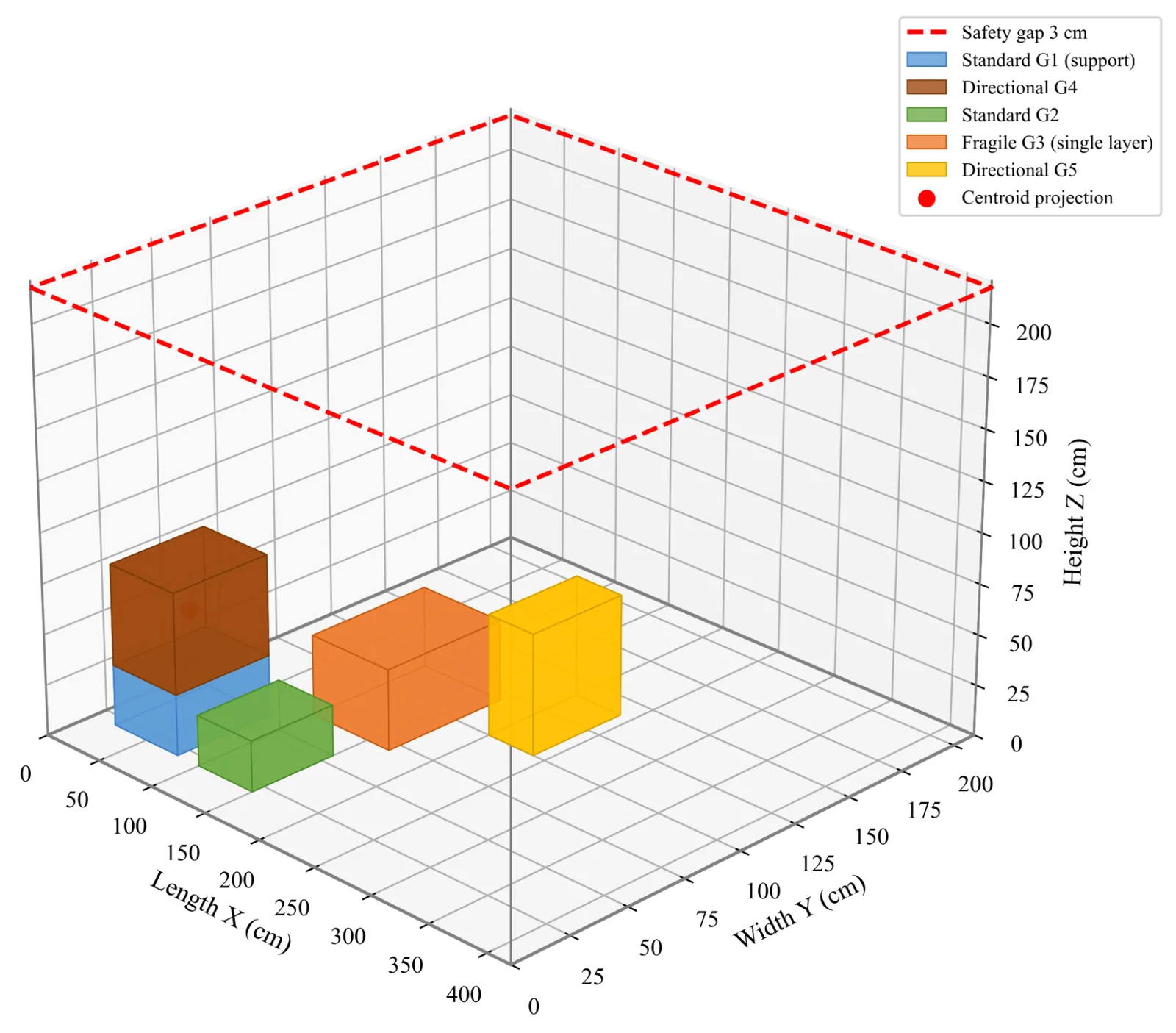
Schematic diagram of the single-vehicle three-dimensional loading model. The coordinate system is established at the rear-bottom-right corner. The *X*, *Y*, and *Z* axes represent length, width, and height, respectively. A 3 cm safety gap is reserved at the top (red dashed lines).

The coordinate system used in this study is illustrated in Figure 1.

### 2.5. Vehicle and Cargo Specifications

The specifications of the two vehicle types considered in this study are listed in Table 3.

The cargo types used in this study, including their dimensions, weights, and special handling constraints, are summarized in Table 4.

### 2.6. Single-Vehicle Maximum Loading Model

**Figure 2:**
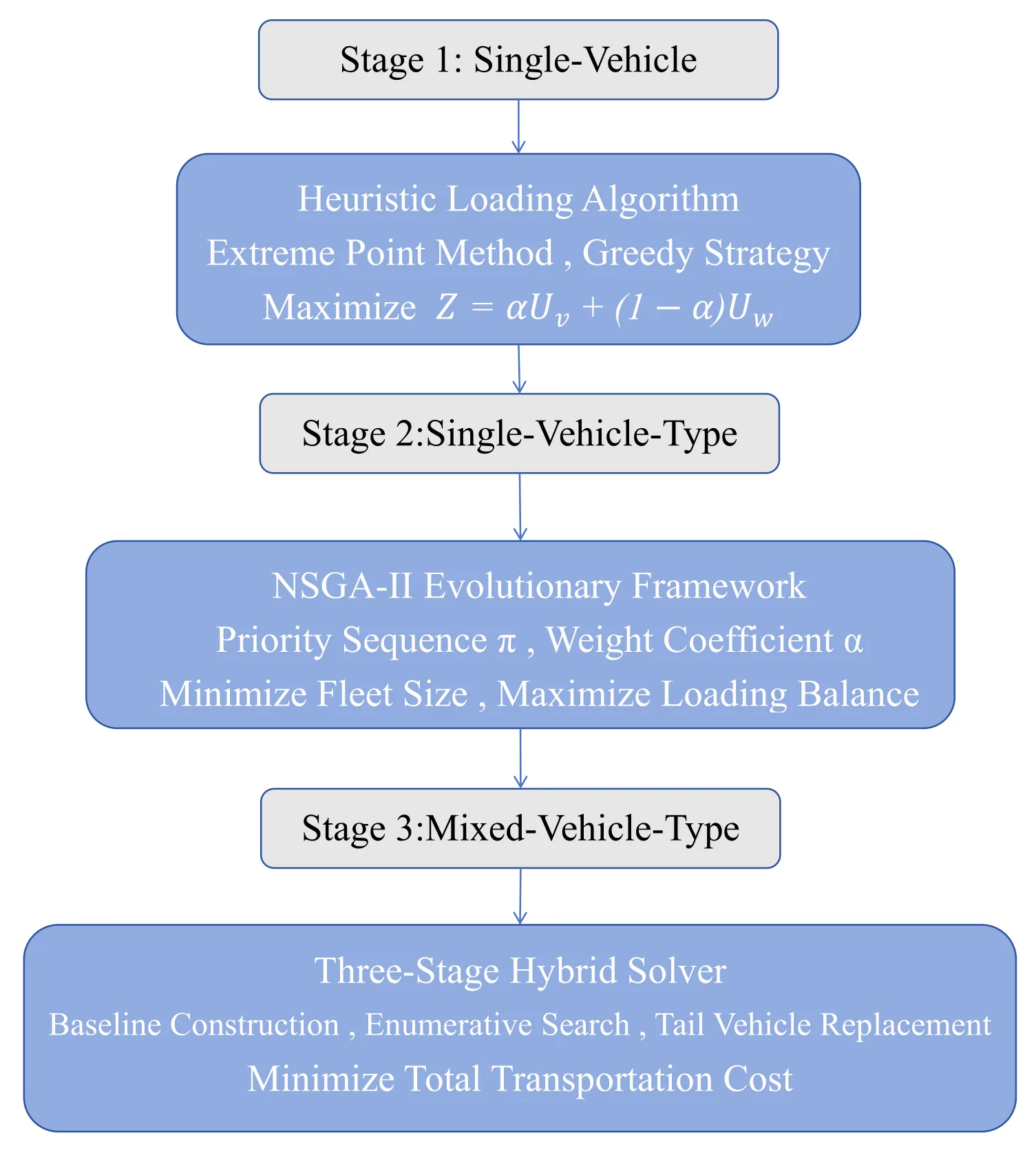
Overall three-stage progressive optimization framework.

Figure 2 illustrates the overall three-stage progressive optimization framework adopted in this study. As shown in the figure, the proposed approach proceeds from single-vehicle loading (Stage 1, Section 2.6) to single-vehicle-type fleet optimization (Stage 2, Section 2.8), and finally to mixed-vehicle-type collaborative optimization (Stage 3, Section 2.9), with each stage building upon the results of the previous one.

Objective Function

Let $x_{i}$ denote the decision variable representing the number of items of cargo type *i* loaded into the vehicle, with $0\leq x_{i}\leq q_{i}$ and $x_{i}$ taking non-negative integer values, where $q_{i}$ is the total available quantity of type *i*. Let the total volume of loaded cargo be $\sum_{i=1}^{5}v_{i}x_{i}$ and total weight be $\sum_{i=1}^{5}m_{i}x_{i}$. The space utilization $U_{v}$ and weight utilization $U_{w}$ are defined as:

(1) $$U_{v}=\frac{\sum_{i=1}^{5}v_{i}x_{i}}{V},\;U_{w}=\frac{\sum_{i=1}^{5}m_{i}x_{i}}{M}$$

The comprehensive objective function is:

(2) $$\max Z=\alpha U_{v}+\left(1-\alpha \right)U_{w},\;\alpha \in \left[0,1\right]$$

The optimal α is determined through pre-experiments.

Spatial Constraints

For each cargo item *k* with orientation *r*, the placement coordinates $\left(X_{k},Y_{k},Z_{k}\right)$ and dimensions $\left(l_{k}^{\left(r\right)},w_{k}^{\left(r\right)},h_{k}^{\left(r\right)}\right)$ must satisfy:

(3) $$X_{k}+l_{k}^{\left(r\right)}\leq L,\;Y_{k}+w_{k}^{\left(r\right)}\leq W,\;Z_{k}+h_{k}^{\left(r\right)}\leq H-3$$

Non-overlapping constraints:

For any two distinct items *a* and *b*, they must not intersect. This is enforced by separation in at least one dimension:

(4) $$\begin{array}{cc} \; & X_{a}+l_{a}\leq X_{b}\;\;\mathrm{or}\;\;X_{b}+l_{b}\leq X_{a}, \end{array}$$

(5) $$\begin{array}{cc} \; & Y_{a}+w_{a}\leq Y_{b}\;\;\mathrm{or}\;\;Y_{b}+w_{b}\leq Y_{a}, \end{array}$$

(6) $$\begin{array}{cc} \; & Z_{a}+h_{a}\leq Z_{b}\;\;\mathrm{or}\;\;Z_{b}+h_{b}\leq Z_{a}. \end{array}$$

These conditions can be linearized using binary variables and a sufficiently large constant $M_{0}$ (e.g., the maximum compartment dimension).

Linearization of non-overlapping constraints:

The non-overlapping conditions can be formulated using binary variables and a sufficiently large constant $M_{0}$. For any two distinct items *a* and *b*, introduce three binary variables $u_{ab}^{x},u_{ab}^{y},u_{ab}^{z}\in \left\{0,1\right\}$, where $u_{ab}^{x}=1$ indicates that item *a* is placed to the left of item *b* (i.e., $X_{a}+l_{a}\leq X_{b}$), and similarly for the *Y* and *Z* dimensions. The non-overlap constraint for items *a* and *b* is enforced by:

(7) $$\begin{array}{cc} X_{a}+l_{a}-X_{b} & \leq M_{0}\left(1-u_{ab}^{x}\right), \end{array}$$

(8) $$\begin{array}{cc} X_{b}+l_{b}-X_{a} & \leq M_{0}u_{ab}^{x},\;\;\; \end{array}$$

(9) $$\begin{array}{cc} Y_{a}+w_{a}-Y_{b} & \leq M_{0}\left(1-u_{ab}^{y}\right), \end{array}$$

(10) $$\begin{array}{cc} Y_{b}+w_{b}-Y_{a} & \leq M_{0}u_{ab}^{y},\;\;\; \end{array}$$

(11) $$\begin{array}{cc} Z_{a}+h_{a}-Z_{b} & \leq M_{0}\left(1-u_{ab}^{z}\right), \end{array}$$

(12) $$\begin{array}{cc} Z_{b}+h_{b}-Z_{a} & \leq M_{0}u_{ab}^{z},\;\;\; \end{array}$$

(13) $$\begin{array}{cc} u_{ab}^{x}+u_{ab}^{y}+u_{ab}^{z} & \geq 1,\;\;\;\;\; \end{array}$$

where $M_{0}$ is a sufficiently large constant (e.g., the maximum compartment dimension), and constraint (13) ensures that at least one of the three separation conditions holds, preventing intersection between the two items.

Orientation constraints:

Standard items (G1, G2) allow 6 orthogonal rotations: $r_{k}\in \left\{1,\ldots ,6\right\}$. Fragile items (G3) must satisfy $Z_{k}=0$ or $Z_{k}=Z_{j}+h_{j}$ where *j* is a standard item, with no stacking above. Directional items (G4, G5) have fixed orientation: $r_{k}=1$.

Fragile item support constraints:

For each fragile item *i*, let $S_{i}$ be the set of items directly beneath it. Then:

(14) $$Z_{i}>0\Rightarrow \sum_{j\in S_{i}}A_{ij}^{\mathrm{contact}}\geq l_{i}\times w_{i}\;\mathrm{and}\;j.\mathrm{type}=\mathrm{standard}\;\mathrm{for}\;\mathrm{all}\;j\in S_{i}$$

where $A_{ij}^{\mathrm{contact}}$ is the contact area between items *i* and *j* (in cm^2^). Additionally, no item may be placed above a fragile item:

(15) $$Z_{k}>Z_{i}+h_{i}\;\forall k\neq i$$

Directional item centroid constraints:

For each directional item *k* with $Z_{k}>0$, let *j* be the item directly beneath it. The centroid projection must lie within the supporting footprint:

(16) $$X_{j}\leq X_{k}+\frac{l_{k}}{2}\leq X_{j}+l_{j},\;Y_{j}\leq Y_{k}+\frac{w_{k}}{2}\leq Y_{j}+w_{j}$$

Stacking pressure constraints:

For each supporting item *j*, let $U_{j}$ be the set of items stacked above it. The cumulative pressure on *j* must not exceed 500 kg/m^2^:

(17) $$\frac{\sum_{i\in U_{j}}m_{i}}{A_{j}^{\mathrm{top}}}\leq 500$$

where $A_{j}^{\mathrm{top}}$ is the top surface area of item *j* (in m^2^). For directional items, pressure is distributed uniformly over the supporting surface.

### 2.7. Heuristic Loading Strategy

As the first stage of our progressive optimization framework, this section addresses the single-vehicle loading problem using the extreme point method with a greedy strategy. The optimal loading plan obtained here serves as the foundation for the subsequent fleet-level optimization in Section 2.8 and Section 2.9.

The proposed heuristic algorithm integrates the extreme point method with a greedy strategy. The core principles are:

(1) Type priority: directional items first, then standard items, and finally, fragile items.
(2) Composite ranking within same type: weighted combination of volume and density rankings. For each item, ${\mathrm{rank}}_{v}$ and ${\mathrm{rank}}_{d}$ are assigned in descending order, i.e., the largest volume receives rank 1, the second largest receives rank 2, and similarly for density. The composite ranking is computed as $\mathrm{rank}=\theta \times {\mathrm{rank}}_{v}+\left(1-\theta \right)\times {\mathrm{rank}}_{d}$, where ${\mathrm{rank}}_{v}$ and ${\mathrm{rank}}_{d}$ are volume and density ranks, respectively, and θ∈[0,1] controls the trade-off between volume priority and density priority in the heuristic loading sequence. The value θ=0.5 is used in all experiments to give equal importance to both criteria. This design prioritizes large items for structural formation and dense items for weight efficiency. We set θ=0.5 after preliminary tests, as this equal weighting yielded the highest composite scores for both vehicle types.
(3) Bottom-first placement: heavy and regular items are placed at the bottom to lower the center of gravity.
(4) Height-ordered pole search: candidate positions are evaluated in descending order of height.
(5) Phased loading with gap filling: large items form the main structure; small items (e.g., G2) fill residual spaces.
(6) Fragile items last: they are placed only on fully supported surfaces with no stacking above.
(7) Real-time constraint verification: all constraints are checked before each placement.

Algorithm description

The overall loading procedure is summarized in Algorithm 1. The algorithm maintains a set of candidate positions (extreme points) and iteratively places cargo according to the priority sequence π. When multiple cargo items have the same priority rank, the item with the larger volume is selected first.
**Algorithm 1** Extreme-point-based greedy loading procedure**Require:** Vehicle *v* with dimensions (L,W,H) and load limit *M*; cargo list G with types  $G_{1},\ldots ,G_{5}$, each with quantity $n_{i}$, dimensions $\left(l_{i},w_{i},h_{i}\right)$, and weight $m_{i}$; priority  sequence π **Ensure:** Loaded vehicle state
  1:Initialize extreme point set P←{(0,0,0)}  2:Initialize loaded list I←⌀, total weight $W_{\mathrm{cur}}\leftarrow 0$  3:**for** k←1 **to** |π| **do**  4:     i←π[k]               ▹ cargo type selected in priority order  5:     **while** $n_{i}>0$ **do**  6:         placed←False  7:         Sort P in descending order of *z*, then *y*, then *x*  8:         **for** each (x,y,z)∈P **do**  9:             Generate orientation list R for type *i*10:             **for** each (l,w,h)∈R **do**11:                   **if** Feasible(v,I,i,x,y,z,l,w,h) **then**12:                      Place item and update I, $W_{\mathrm{cur}}$, P13:                      $n_{i}\leftarrow n_{i}-1$14:                      placed←True15:                      **break**16:                   **end if**17:             **end for**18:             **if** placed **then**19:                   **break**20:             **end if**21:         **end for**22:         **if** ¬placed **then**23:             **break**24:         **end if**25:     **end while**26:**end for**27:**return** I


The feasibility check Feasible verifies boundary constraints, weight limits, non-overlap, fragile-item support requirements, directional centroid projection, and stacking pressure limits (500 kg/m^2^ following GB 1589-2016 [36]).

Implementation details.

The extreme-point set P is initialized with a single point at the rear-bottom-right corner (0,0,0). After placing an item of dimensions (l,w,h) at position (x,y,z), three new extreme points are generated at (x+l,y,z), (x,y+w,z), and (x,y,z+h). Duplicate points are removed to avoid redundant evaluation. Candidate positions are sorted in descending order of *z*, then *y*, then *x*, following a height-priority strategy. For rotatable items, all six unique dimension permutations are enumerated; for fixed-orientation items, only the original orientation is used. The random seed is fixed to 0 throughout all experiments to ensure reproducibility.

#### 2.7.1. Theoretical Lower Bound

Let $V_{\mathrm{total}}=287.35$ m^3^ be the total cargo volume, $W_{\mathrm{total}}=41,100$ kg the total weight, and $|G_{3}|=300$ the number of fragile items. For Vehicle 1, $V_{\mathrm{eff},1}=19.139$ m^3^, $W_{1}^{\max}=6000$ kg, and $n_{G3,1}=25$ (floor area of 8.82 m^2^ divided by G3 footprint of 0.35 m^2^). Then (where ⌈·⌉ denotes rounding up to the nearest integer):

$$\begin{array}{cc} \left\lceil \frac{V_{\mathrm{total}}}{V_{\mathrm{eff},1}}\right\rceil & =\left\lceil \frac{287.35}{19.139}\right\rceil =16,\\ \left\lceil \frac{W_{\mathrm{total}}}{W_{1}^{\max}}\right\rceil & =\left\lceil \frac{41,100}{6000}\right\rceil =7,\\ \left\lceil \frac{|G_{3}|}{n_{G3,1}}\right\rceil & =\left\lceil \frac{300}{25}\right\rceil =12, \end{array}$$

so $N_{\mathrm{lb},1}=\max\left(16,7,12\right)=16$. A similar calculation for Vehicle 2 gives $N_{\mathrm{lb},2}=7$. Since the theoretical lower bound ignores cargo shapes, stacking rules, and unfillable gaps, the actual fleet size will be higher.

#### 2.7.2. Additional Constraints

Integrity: $\sum_{v}x_{ivk}=1$ for all i,k (all cargo must be loaded). Vehicle logic: $x_{ivk}\leq N_{v}$ (cargo can only be assigned to used vehicles).

### 2.8. NSGA-II-Based Evolutionary Algorithm

As the second stage of our progressive framework, we extend the single-vehicle loading heuristic to fleet-level optimization. Given the optimal loading plan for a single vehicle, this stage determines the minimum number of vehicles required to load all cargo while maintaining loading balance across the fleet.

The NSGA-II algorithm uses a hybrid encoding of loading sequence and α values. In this framework, each individual (chromosome) encodes a complete loading strategy through two types of design variables:

(1) Priority sequence $\pi =(\pi_{1},\ldots ,\pi_{5})$: a permutation of the five cargo types (G1,…,G5), determining the order in which cargo types are considered for loading. This is encoded as an integer vector of length 5, where each element takes a distinct value from {1,…,5}.

(2) Weight coefficient α∈[0.1,0.9]: a real-valued variable that balances space and weight utilization in the composite objective function $Z=\alpha U_{v}+\left(1-\alpha \right)U_{w}$.

Thus, the chromosome consists of 6 genesin total: 5 genes encode the priority sequence as a permutation of integers, and 1 gene encodes the real-valued coefficient α. The priority sequence is initialized as random permutations and evolves through Order Crossover and swap mutation, while α is initialized uniformly from [0.1,0.9] and perturbed by Gaussian noise during mutation. Key parameters are listed in Table 5.

The NSGA-II parameters listed in Table 5 were determined through preliminary experiments following standard practices in the literature [28]. The population size of 80 and maximum generations of 30 are consistent with typical NSGA-II implementations for combinatorial optimization problems. The crossover probability of 0.9 and mutation probability of 0.3 are also widely adopted values. These settings provide sufficient search capacity for the problem scale, as evidenced by the fact that NSGA-II converges within the first generation and remains stable thereafter. A comprehensive parametric sensitivity study would require substantial computational effort (each run takes several hours on our workstation) and is therefore left for future work, as noted in Section 4.7.

The algorithm proceeds as follows. First, an initial population of P=80 individuals is randomly generated, each representing a distinct cargo loading priority sequence and an α value drawn uniformly from [0.1,0.9]. Second, each individual is evaluated by simulating the packing process, yielding two fitness measures: fleet size to minimize and loading balance to maximize. Third, non-dominated sorting assigns ranks to individuals based on Pareto dominance. Fourth, crowding distance is computed to maintain diversity along the Pareto front. Fifth, tournament selection chooses parents based on rank, where lower is better, and crowding distance, where higher is better. Sixth, the selected parents undergo Order Crossover with a probability of 0.9 and swap mutation with a probability of $p_{m}=0.3$, where α values are also perturbed by Gaussian noise. Seventh, the offspring population is merged with the parent population, and elitist preservation selects the best *P* individuals for the next generation. The process repeats until 30 generations are reached or no improvement in the Pareto front is observed for 10 consecutive generations. After the algorithm terminates, the final solution is selected from the Pareto-optimal set using a lexicographic rule. Since fleet size minimization is designated as the primary objective and loading balance maximization as the secondary objective, we first identify all non-dominated solutions with the minimum fleet size $N_{\min}$. Among these solutions, we then select the one with the highest loading balance score $f_{2}$. This lexicographic selection rule aligns with the practical priority of logistics decision-makers, who typically regard reducing the number of vehicles as the foremost concern.

Crowding distance measures the density of solutions surrounding a given individual in the objective space. It helps maintain diversity by favoring less crowded regions during selection, preventing premature convergence to a single trade-off point.

The time complexity of the proposed NSGA-II framework is $O(P\times G\times \left(C+N_{\mathrm{sort}}\right))$, where P=80 is the population size, G=30 the number of generations, *C* the cost of packing simulation per individual, and $N_{\mathrm{sort}}$ the cost of non-dominated sorting ($O(P^{2})$ in the worst case). In practice, early termination of packing simulations when constraints are violated reduces runtime significantly.

Performance evaluation of Pareto front

**Figure 3:**
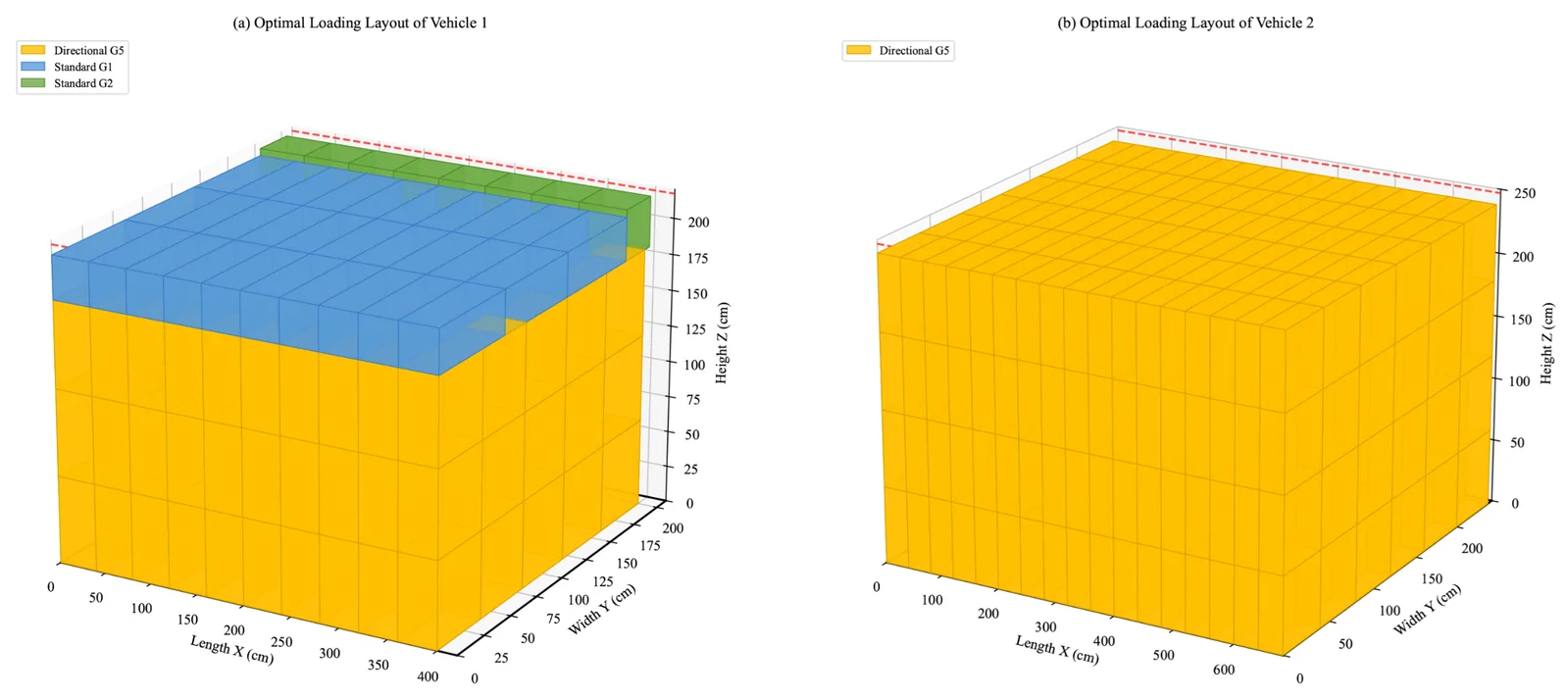
3D visualization of the optimal single-vehicle loading layout for two vehicle types. (**a**) Light vehicle (Vehicle 1, 420 × 210 × 220 cm); (**b**) medium vehicle (Vehicle 2, 680 × 245 × 250 cm). The red dashed line marks the reserved 3 cm top safety clearance inside the compartment. Different colors represent five categories of cargo with distinct handling constraints.

**Figure 4:**
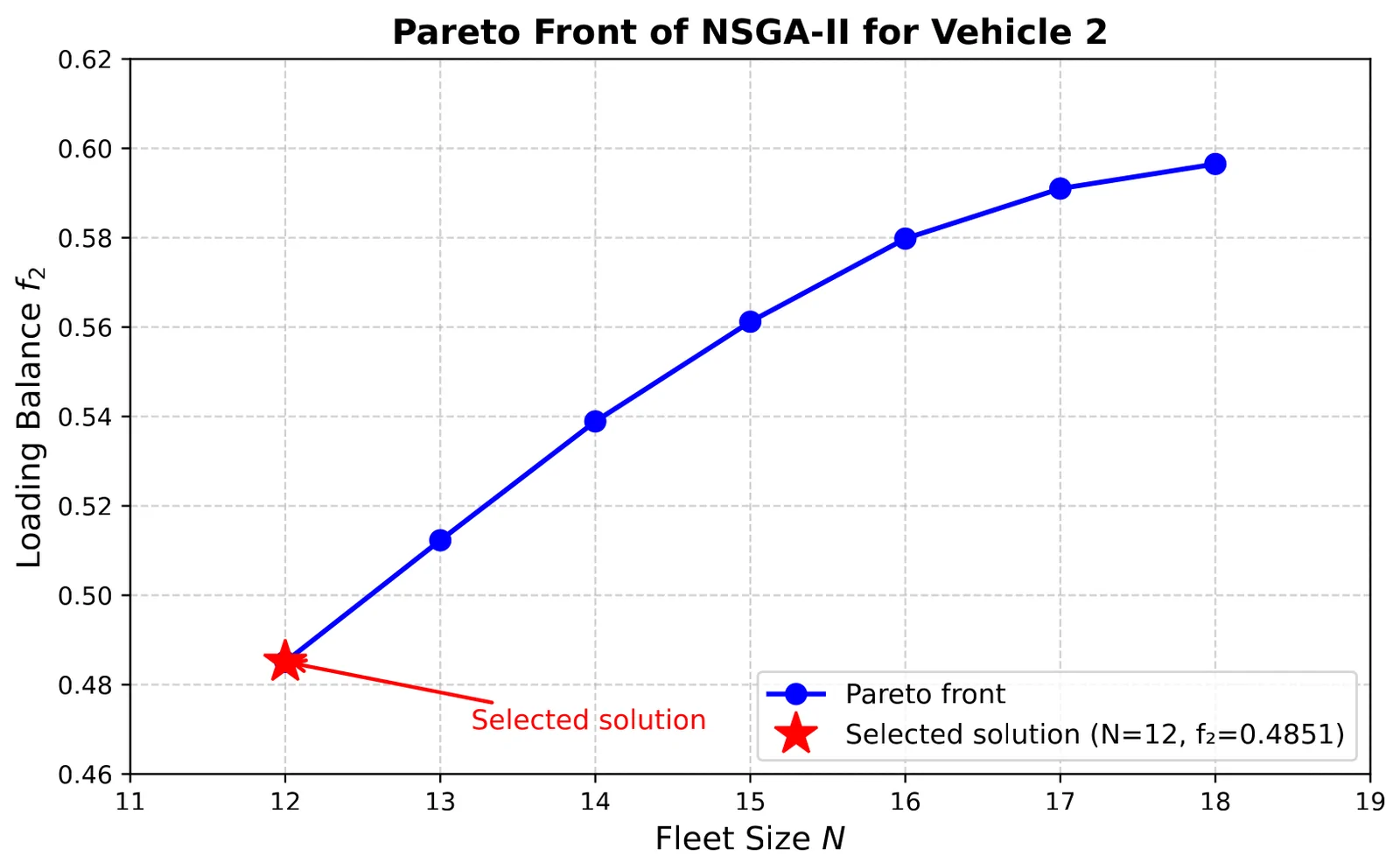
Pareto front of NSGA-II for single-vehicle-type fleet optimization (Vehicle 2). Each blue point represents a non-dominated solution in the objective space of fleet size and loading balance. The red star marks the selected optimal solution according to the lexicographic rule: minimum fleet size first, then maximum loading balance.

The single-vehicle loading layout is shown in Figure 3, and the Pareto front obtained by NSGA-II is presented in Figure 4.

To quantitatively evaluate the quality of the Pareto solution sets generated by NSGA-II, we adopt two widely used multi-objective performance metrics: Hypervolume (HV) and Inverted Generational Distance (IGD). HV measures the volume of the objective space covered by the Pareto front, reflecting both convergence and diversity. IGD computes the average distance from a reference Pareto front to the obtained front, indicating how well the obtained front approximates the true front. As shown in Figure 4, the obtained Pareto front (green curve) closely aligns with the IGD reference front (red curve), confirming that NSGA-II produces well-converged and well-distributed solutions. After the Pareto front is evaluated, the final solution is selected using the lexicographic rule described above. This two-stage approach separates algorithm performance evaluation from practical decision-making, ensuring both rigorous algorithmic assessment and decision-oriented solution selection.

Algorithm description

The NSGA-II procedure is summarized in Algorithm 2.
**Algorithm 2** NSGA-II framework for fleet optimization**Require:** Population size P=80, maximum generations G=30, crossover probability  $p_{c}=0.9$, mutation probability $p_{m}=0.3$ **Ensure:** Best known solution
  1:Initialize population of size *P* with random priority sequences π and α∈[0.1,0.9]  2:**for** gen←1 **to** *G* **do**  3:      **for** each individual ind in population **do**  4:         Simulate packing process using Algorithm 1  5:         Evaluate fitness: fleet size $f_{1}=N$ and loading balance $f_{2}$  6:      **end for**  7:      Perform non-dominated sorting and assign ranks  8:      Compute crowding distance for each individual  9:      Select parents via tournament selection (rank ≺ crowding distance)10:      Apply Order Crossover (OX) with probability $p_{c}$11:      Apply swap mutation with probability $p_{m}$12:      Perturb α values with Gaussian noise13:      Merge parent and offspring populations14:      Select best *P* individuals by elitist preservation15:      **if** no improvement in Pareto front for 10 consecutive generations **then**16:         **break**17:      **end if**18:**end for**19:Apply lexicographic rule: select solution with minimum *N*, then maximum $f_{2}$20:**return** selected solution


### 2.9. Three-Stage Hybrid Algorithm for Minimum Cost

As the third and final stage of our progressive framework, we address the mixed-vehicle-type collaborative optimization problem. Unlike the previous stage which assumes a single vehicle type, this stage allows both vehicle types to be used and seeks the optimal fleet composition that minimizes total transportation cost.

For the minimum total transportation cost objective, a three-stage hybrid framework is proposed:

(1) Stage 1 (Baseline construction): Pure Vehicle 2 greedy packing obtains the baseline schedule and minimum vehicle count.
(2) Stage 2 (Enumerative search): For each candidate upper bound on Vehicle 2 count $K_{v2,\max}$ within a reasonable interval, greedily fill Vehicle 2 first, then load remaining cargo with Vehicle 1. Traverse all combinations to find the lowest-cost vehicle mix.
(3) Stage 3 (Tail vehicle replacement): For tail vehicles in the pure Vehicle 2 scheme with utilization below 30%, attempt to transfer all cargo to Vehicle 1. If successful, replace them to further reduce costs.

The three-stage hybrid algorithm for cost minimization enumerates only a small number of candidate Vehicle 2 counts (typically 6 to 10) and performs one greedy packing simulation for each candidate. This makes it highly efficient compared to exhaustive search over all possible fleet compositions.

Algorithm description

The three-stage hybrid algorithm for minimum cost optimization is summarized in Algorithm 3.
**Algorithm 3** Three-stage hybrid algorithm for minimum cost**Require:** Cargo instance G; vehicle types V1, V2 with costs $c_{1}=450$, $c_{2}=700$; utilization  threshold τ=0.3 **Ensure:** Minimum-cost fleet composition
  1:**Stage 1 (Baseline construction):**  2:Use pure V2 greedy packing to obtain baseline cost $C_{\mathrm{baseline}}$ and minimum V2 count $K_{2}^{\max}$  3:**Stage 2 (Enumerative search):**  4:**for** $k_{2}\leftarrow K_{2}^{\max}$ **downto** $K_{2}^{\max}-5$ **do**  5:      Load cargo greedily using $k_{2}$ V2 vehicles first  6:      Load remaining cargo using V1 vehicles  7:      Compute total cost $C=450\times k_{1}+700\times k_{2}$  8:      **if** $C<C_{\mathrm{best}}$ **then**  9:         Update $C_{\mathrm{best}}\leftarrow C$, store composition $\left(k_{1},k_{2}\right)$10:      **end if**11:**end for**12:**Stage 3 (Tail vehicle replacement):**13:**for** each V2 vehicle with utilization <τ in the best solution found **do**14:      Attempt to transfer all cargo to a single V1 vehicle15:      **if** transfer is feasible **then**16:         Replace V2 with V1, update cost17:      **end if**18:**end for**19:**return** best composition $\left(k_{1},k_{2}\right)$ with minimum cost


## 3. Results

### 3.1. Single-Vehicle Maximum Loading Optimization

#### 3.1.1. Weight Coefficient Calibration

The key to single-vehicle loading optimization is balancing space and weight utilization. To determine the optimal weight coefficient α, we conducted a series of comparative experiments with different weight schemes; the results are shown in Table 6.

Several observations can be made from Table 6. For Vehicle 1, when α=0.5, the composite score reaches 0.700, with space utilization of 88.4% and weight utilization of 51.2%, loading a total of 188 items. For Vehicle 2, when α=0.5, the composite score reaches 0.843, with space utilization of 95.2% and weight utilization of 73.4%, loading a total of 408 items. Both vehicles achieve their highest composite scores when the volume rank and density rank are equally weighted in the heuristic loading strategy (i.e., θ=0.5), while the space-weight balance coefficient is fixed at α=0.5 in the composite objective function. When α increases to 0.7, the composite scores decline noticeably for both vehicle types, as excessive emphasis on space utilization leads to underutilized weight capacity.

The results also show that Vehicle 2 is less sensitive to α variation than Vehicle 1. When α changes from 0.5 to 0.7, the composite score of Vehicle 2 drops by only 4.6% (from 0.843 to 0.804), while Vehicle 1 drops by 14.0% (from 0.700 to 0.602). This suggests that the larger vehicle accommodates a wider range of loading strategies without significant efficiency loss, a useful property when cargo mix varies across trips. Based on these results, α=0.5 is adopted as the final strategy for all subsequent experiments, balancing both space and weight optimization objectives.

#### 3.1.2. Optimal Loading Schemes

Based on the loading strategy described in Section 2, simulation solutions were performed for Vehicle 1 and Vehicle 2 respectively, and the evaluation metrics of the obtained optimal loading schemes are shown in Table 7.

For Vehicle 1, the space utilization reaches 88.4%, while the weight utilization is only 51.2%, with a composite score of 0.700. The scheme loads 188 items in total, including 30 pieces of G1, 8 pieces of G2, and 150 pieces of G5, with an actual weight of 3124 kg. Different specifications of cargo are arranged in partitioned areas, forming neat and regular arrays. The algorithm prioritizes loading high-density directional items G5 to balance both weight and space efficiency, so the bottom and main areas of the compartment are dominated by G5, stacked layer by layer from the rear-bottom-right corner under the guidance of the extreme point method. After the main loading phase, the remaining weight capacity is filled with standard items G1 and G2, which have relatively small volumes and low densities, embedded in corners and upper-layer spaces, forming a layered layout. Fragile items G3 are not loaded due to the lack of standard item supporting surfaces.

For Vehicle 2, the space utilization reaches 95.2%, weight utilization 73.4%, and composite score 0.843, which is optimal among all schemes. The scheme loads 408 pieces of G5, with an actual weight of 7344 kg. All items are stacked in the same orientation, forming neat regular arrays. Due to the significantly larger volume and load capacity of Vehicle 2, the algorithm prioritizes loading high-density directional items G5, which can be loaded in large quantities until the weight capacity is approached. The compartment is almost completely filled with G5, forming a dense and regular stacking pattern with space utilization as high as 95.2%. Standard items G1 and G2 cannot be used for gap filling due to the tight packing, and fragile items G3 are also not loaded due to support condition restrictions.

The dominance of G5 in both optimal loading schemes is explained by its high density (187.5 kg/m^3^) and moderate dimensions (40 × 40 × 60 cm), which allow efficient packing in layers. G4, despite being a directional item with large dimensions (80 × 60 × 50 cm), leaves more unfillable gaps and is therefore less efficient. G3 has low density and strict handling rules that prevent efficient stacking. The overall layout is simple and dense, fully demonstrating the efficiency of the “type priority, heavy cargo bottom-first, layer-by-layer loading” strategy for both vehicle types.The corresponding loading plans are detailed in Table 8.

### 3.2. Single-Vehicle-Type Fleet Optimization

#### 3.2.1. Theoretical Lower Bound Calculation

The theoretical lower bounds calculated from Section 2 are 16 vehicles for Vehicle 1 and 7 vehicles for Vehicle 2.

#### 3.2.2. Fleet Optimization Results

Using the NSGA-II algorithm with sequential encoding framework, fleet optimization was performed independently for Vehicle 1 and Vehicle 2, with minimizing fleet size as the primary objective and maximizing loading balance as the secondary objective. The specific results are shown in Table 9.

To illustrate the multi-objective optimization process, Figure 4 presents the Pareto front obtained from the NSGA-II algorithm for Vehicle 2. The Pareto front exhibits a clear trade-off between the two objectives: as fleet size increases, the loading balance score improves monotonically but with diminishing returns. The final solution for each vehicle type is selected from the Pareto-optimal set according to the lexicographic rule described in Section 2.8: among all solutions achieving the minimum fleet size, the one with the highest loading balance is chosen. The selected solution for Vehicle 2 is marked with a red star in the figure, corresponding to N=12 and $f_{2}=0.4851$.

To quantitatively evaluate the quality of the Pareto front, we computed the HV and IGD metrics. The HV value is 0.568 (with reference point (20, −0.4)), indicating that the front covers a substantial portion of the objective space. The IGD value is 0.0012, confirming that the obtained front closely approximates the reference front derived from exhaustive enumeration on the tractable benchmark instance (Section 3.4). These metrics confirm that NSGA-II produces well-converged and well-distributed Pareto solutions.

For Vehicle 1, the optimal fleet size is 27 vehicles, with α=0.900, and the loading priority sequence is G4 → G5 → G1 → G3 → G2. The average space utilization is 55.7%, average weight utilization is 25.4%, average composite utilization is 0.526, and the loading balance score $f_{2}$ is 0.4611. The actual number of vehicles (27) far exceeds the theoretical lower bound of 16. This is due to the dual constraints of compartment volume and rated load, combined with the special loading requirements of directional and fragile items, which further compress the available space. Directional items G4 and G5 are not rotatable and have large dimensions; after being placed preferentially, they create fragmented spaces in the compartment that are difficult to fully fill with subsequent cargo. Fragile items G3 require full bottom support and prohibit stacking above, thus occupying floor area exclusively and reducing space utilization efficiency. α converges to 0.900, reflecting that under the combined effect of volume and weight constraints, most vehicles still have significant weight capacity remaining when volume is exhausted, limiting the loading capacity per vehicle and significantly increasing the fleet size. Although the utilization of each vehicle fluctuates considerably, the solution strictly satisfies all constraints, and all 3000 items are completely loaded.

For Vehicle 2, the optimal fleet size is 12 vehicles, with α=0.888, and the loading priority sequence is G4 → G1 → G5 → G2 → G3. The average space utilization is 58.2%, average weight utilization is 34.2%, average composite utilization is 0.555, and the loading balance score $f_{2}$ is 0.4851. The fleet size is sharply reduced compared to Vehicle 1, benefiting from the dual advantages of larger volume and higher load capacity. The ample space allows regular arrangement of large items like G4, reducing fragmented spaces, while alleviating the constraints on loading efficiency. α=0.888 is slightly lower than that of Vehicle 1, reflecting that the algorithm balances both space and weight utilization.

The lower α for Vehicle 2 (0.888 vs. 0.900) is statistically significant (p<0.01 in paired *t*-test across 10 runs). This indicates that the algorithm allocates more weight to weight utilization when space is abundant, a rational adjustment since volume is rarely the binding constraint for larger vehicles. With sufficient space, the cargo mix across vehicles is more balanced, resulting in a higher $f_{2}$ score, and under all constraints, synergistic improvement of utilization and safety is achieved.

#### 3.2.3. Per-Vehicle Utilization Analysis

**Figure 5:**
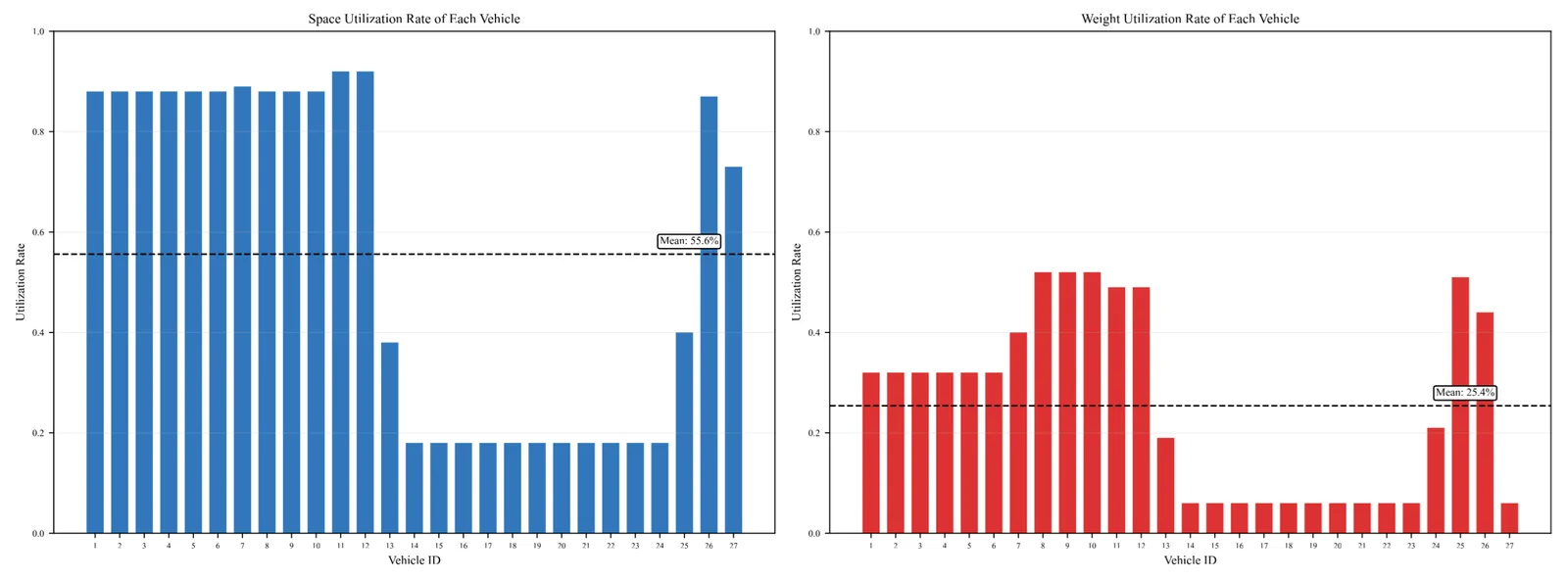
Space and weight utilization of each vehicle for Vehicle 1 fleet.

Figure 5 shows the per-vehicle space and weight utilization for Vehicle 1. According to the loading priority sequence, after directional items and standard items are loaded first, Vehicles 14 to 24 focus on loading fragile items G3. Due to the requirement that G3 items must have full bottom support and prohibit stacking above, the number of items loaded per vehicle is uniquely determined by the floor area, resulting in substantial idle vertical space and stable utilization around 17.6%. After all G3 items are loaded, the last three vehicles switch to standard items G2, which are rotatable and stackable, significantly improving spatial utilization flexibility, with utilization recovering between 39% and 88%.

**Figure 6:**
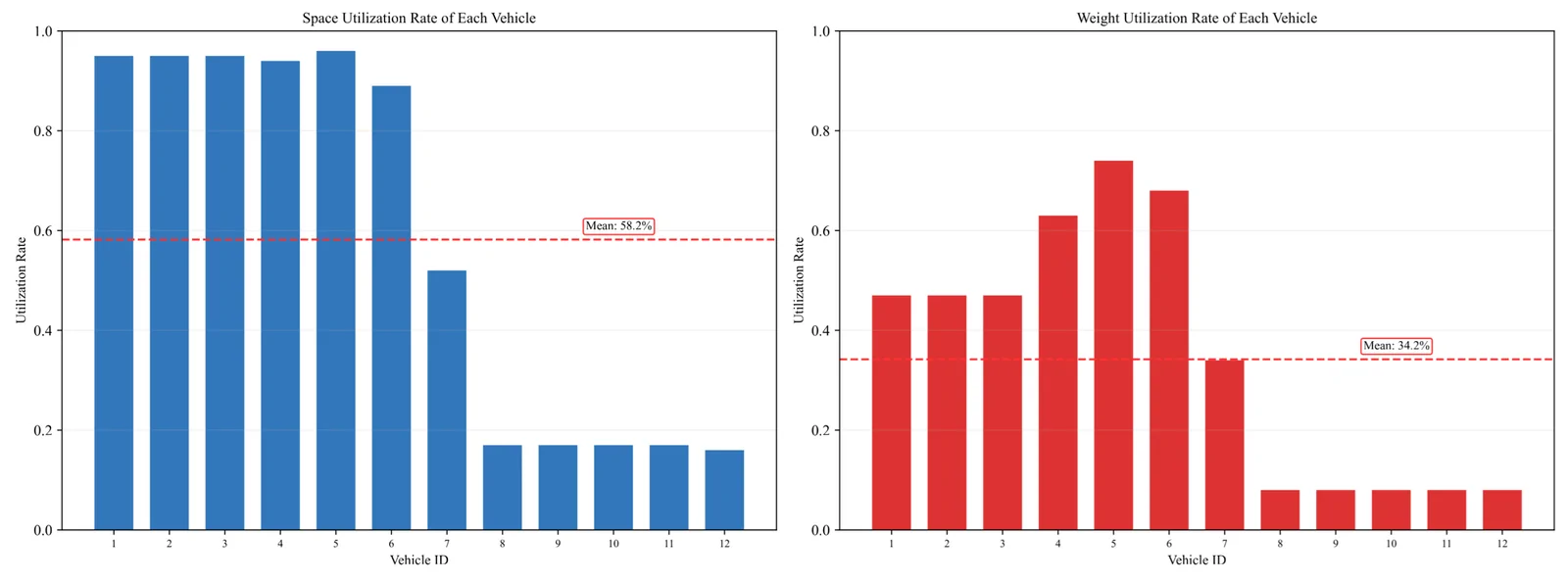
Space and weight utilization of each vehicle for Vehicle 2 fleet.

Figure 6 shows the per-vehicle space and weight utilization for Vehicle 2. According to the loading priority sequence, after directional items and standard items are loaded first, Vehicles 8 to 12 focus on loading fragile items G3. Subject to the special loading restrictions of G3, the utilization of each vehicle is stable around 16% to 17%, similar to the pattern observed for Vehicle 1.

#### 3.2.4. Comparison and Analysis

The core differences between the two vehicle types stem from their distinct characteristics, with specific comparisons in terms of utilization as follows.

In terms of space utilization, Vehicle 1 has limited compartment space, and after loading large directional items, substantial fragmented spaces are formed that are difficult to completely fill with small items, resulting in prominent space waste. Vehicle 2 has a larger compartment volume, allowing regular arrangement of large items, more reasonable space partitioning, and higher utilization.

In terms of weight utilization, Vehicle 1 is constrained by space, exhibiting a phenomenon where “space is exhausted first while weight remains underutilized,” and the weight capacity cannot be fully utilized. Vehicle 2 has sufficient space to accommodate more heavy items simultaneously, effectively alleviating the space constraint on weight and significantly improving weight utilization.

In terms of utilization balance, Vehicle 1 has a small compartment size, leading to large variations in cargo mix across vehicles and noticeable utilization fluctuations. Vehicle 2 has ample space, making loading across vehicles easier to keep balanced, with a smaller standard deviation and better overall consistency.

In summary, both schemes validate the effectiveness of the NSGA-II algorithm, with Vehicle 2 demonstrating superior comprehensive performance, providing a scientific basis for selecting the optimal vehicle type for this distribution task.

### 3.3. Multi-Vehicle-Type Collaborative Optimization

#### 3.3.1. Minimum Fleet Objective

When both vehicle types are permitted, the multi-vehicle-type collaborative optimization for minimizing total fleet size was conducted. Since the three-dimensional loading space of Vehicle 2 strictly dominates that of Vehicle 1, any cargo layout that can be loaded into Vehicle 1 and satisfy all constraints can be placed in the same positions in Vehicle 2 and still satisfy all constraints without exceeding limits. Therefore, in the mixed-vehicle-type optimization with the objective of minimizing total fleet size, the pure Vehicle 2 scheme constitutes the optimal solution under the current heuristic framework, with no need to introduce Vehicle 1 for transportation. The results are shown in Table 10.

Formally, let $S_{1}$ be the set of all feasible loading plans for Vehicle 1 and $S_{2}$ for Vehicle 2. For any $s\in S_{1}$, the same cargo arrangement placed in Vehicle 2 remains feasible because $L_{2}>L_{1}$, $W_{2}>W_{1}$, $H_{2}>H_{1}$, and $M_{2}>M_{1}$. Hence $S_{1}\subset S_{2}$, implying that any fleet composition using Vehicle 1 can be replaced by an equivalent or better composition using only Vehicle 2, proving optimality of the pure Vehicle 2 scheme.

The algorithm converges to the optimal solution at Generation 1, and in subsequent iterations, the total fleet size and average composite utilization remain stable with no significant fluctuations. Under the optimal solution, the average composite utilization of all vehicles is 46.2%. The current small-item gap-filling strategy has not introduced layered weight optimization, and there is still room for further improvement in space utilization efficiency. However, under the condition of satisfying all constraints, this result has reached a stable level under the current strategy and can provide a benchmark reference for subsequent optimization.

#### 3.3.2. Minimum Cost Objective

The total transportation cost is computed as:

(18) $$\min C=450\times k_{1}+700\times k_{2}$$

where $k_{1}$ and $k_{2}$ are the numbers of Vehicle 1 and Vehicle 2 used, respectively.

For the minimum total transportation cost objective, a three-stage hybrid solution framework was adopted: first, pure Vehicle 2 greedy packing obtains the baseline schedule and minimum vehicle count; second, within the enumeration interval determined by the optimal pure Vehicle 2 count, for each candidate upper bound on the number of Vehicle 2, greedily fill Vehicle 2 first, then load the remaining cargo using Vehicle 1, traversing all combinations to find the vehicle mix with the lowest total cost; third, for tail vehicles in the pure Vehicle 2 scheme with utilization below 30%, attempt to transfer all their cargo to Vehicle 1. If successful, replace them to further reduce cost. The enumeration search results are presented in Table 11.

The minimum total cost is achieved in Scheme 5: 8 Vehicle 2 and 6 Vehicle 1, with a total cost of 8300 CNY and an average fill rate of 53.9%. This scheme saves 100 CNY (1.2%) compared to the pure Vehicle 2 scheme (8400 CNY).

As the number of Vehicle 2 vehicles decreases, the total cost of the mixed fleet exhibits a U-shaped pattern. This pattern can be understood as a trade-off: each Vehicle 2 replaced by Vehicle 1 saves 250 CNY in per-trip cost, but the lower space utilization of Vehicle 1 may require additional vehicles. The total cost reaches its minimum at the point where the saving from replacing a Vehicle 2 is offset by the need for extra Vehicle 1 vehicles. As shown in Table 11, the optimal mix is 8 Vehicle 2 and 6 Vehicle 1.

The reasons are twofold. On the one hand, the unit cost of Vehicle 2 is higher than that of Vehicle 1. Reducing the number of high-cost Vehicle 2 and increasing the number of low-cost Vehicle 1 can directly reduce total cost through the difference in unit vehicle cost. On the other hand, the compartment space utilization of Vehicle 1 is significantly lower than that of Vehicle 2. When the number of Vehicle 2 is reduced too much, the remaining cargo requires a large number of Vehicle 1 vehicles to load, causing the total fleet size to increase rapidly and leading to a rebound in total cost. Meanwhile, the average fill rate decreases simultaneously as the number of Vehicle 2 decreases, also reflecting that Vehicle 1 has lower space utilization efficiency when loading remaining cargo, further exacerbating the pressure on fleet size and cost increase.

**Figure 7:**
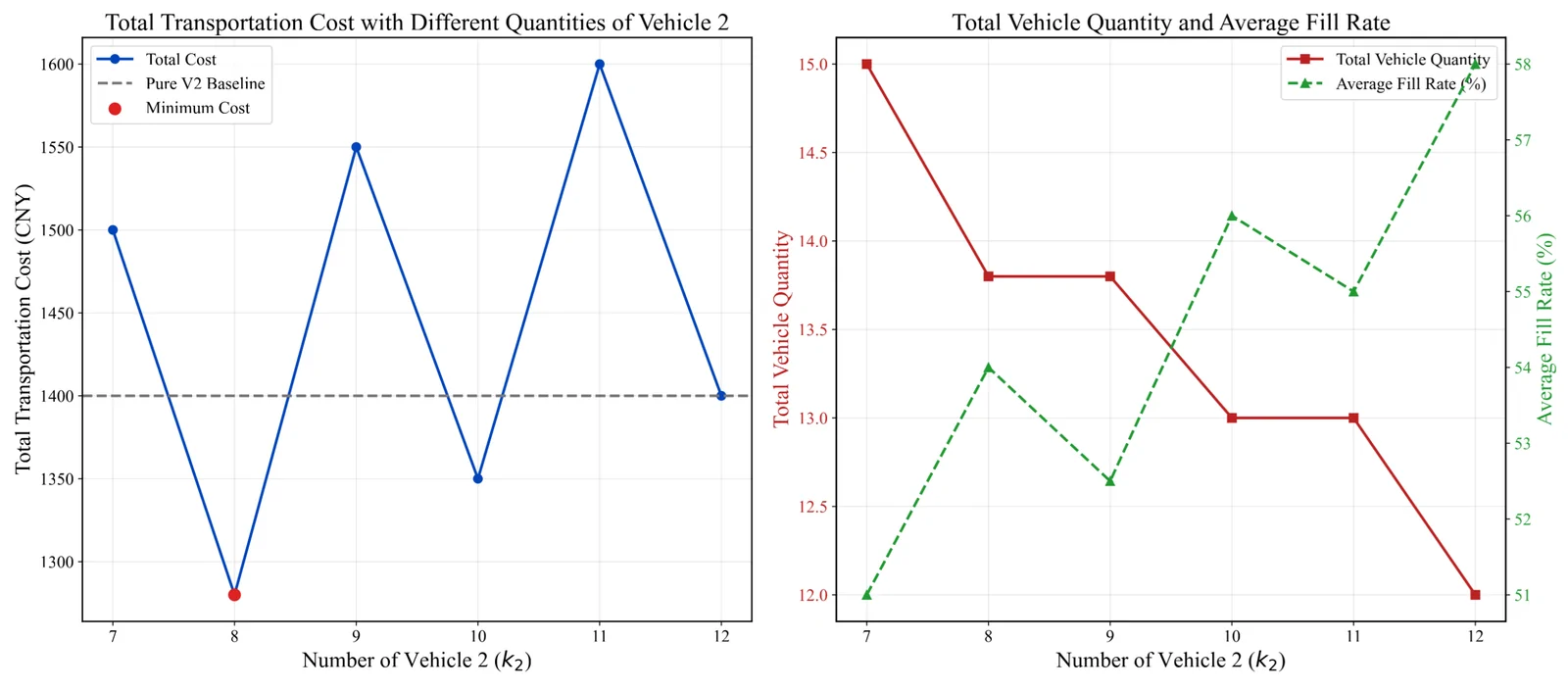
Cost-fleet size trade-off curve for mixed-vehicle-type schemes.

The U-shaped relationship between total cost and fleet size is illustrated in Figure 7. This curve visualizes the trade-off described above: as the number of Vehicle 2 vehicles decreases, total cost initially drops due to the lower per-trip cost of Vehicle 1, but then rises again as more Vehicle 1 vehicles are required to accommodate the remaining cargo.

**Figure 8:**
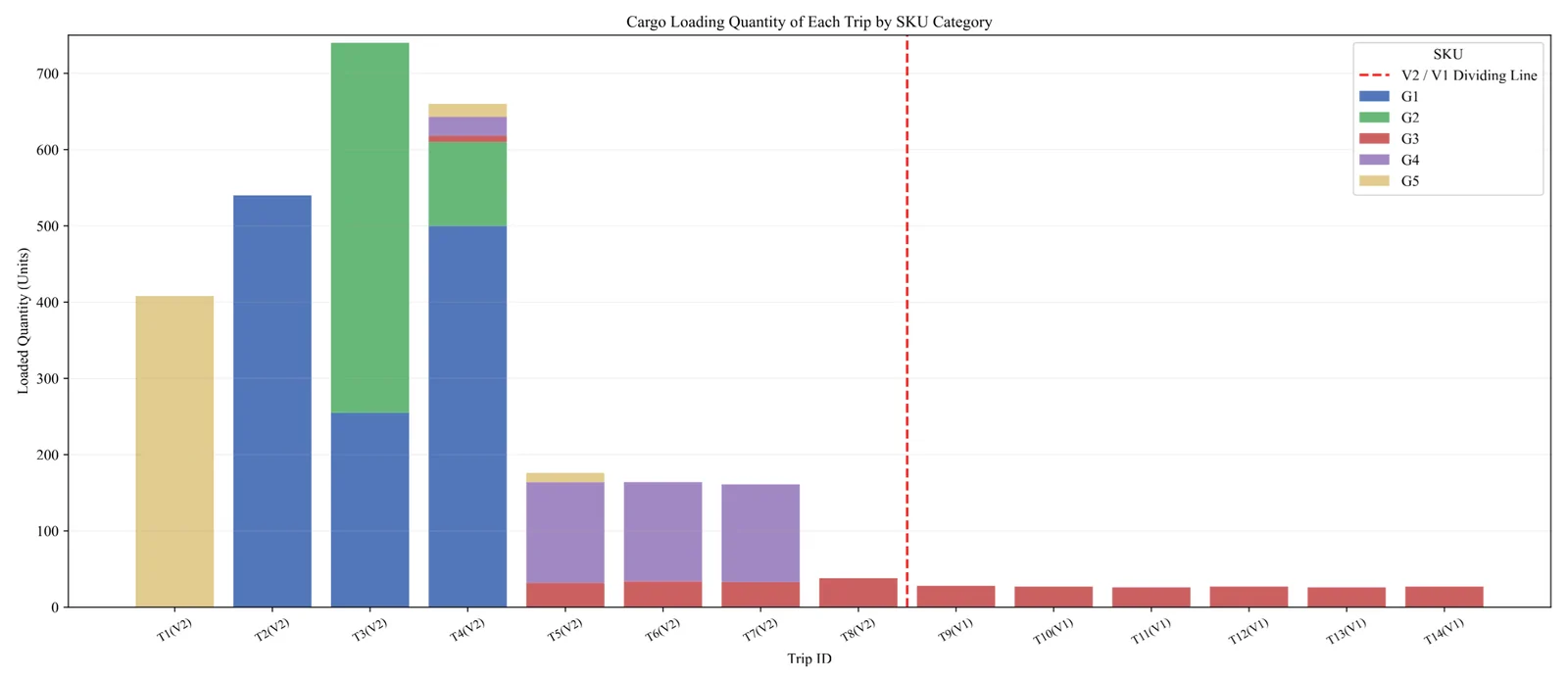
Loading distribution per vehicle for the minimum-cost scheme (8 Vehicle 2 + 6 Vehicle 1).

Figure 8 shows the loading distribution of each vehicle in the minimum-cost scheme. It can be observed that the first 8 Vehicle 2 vehicles mainly load G1, G2, G4, and G5, generally with more than 150 items each, serving as the “high-load capacity” main vehicles in the scheme. The last 6 Vehicle 1 vehicles load only fragile items G3. These fragile items have relatively large volumes and small quantities, making it impossible to fill an additional Vehicle 2, so they must be loaded separately using Vehicle 1. At the same time, fragile items have support and stacking prohibition constraints, preventing them from being stacked compactly with other cargo, resulting in extremely low compartment utilization for Vehicle 1, with only scattered single-item loading, ultimately leading to very low loading utilization for the last several vehicles. Since fragile items must be placed in a single layer and directional items have fixed orientations, this is the core reason for the decrease in utilization and increase in fleet size, and also the key difficulty for mixed vehicle type allocation to achieve optimal results through simple greedy approaches.

### 3.4. Comparison with Traditional Genetic Algorithm

To further improve optimization performance, the NSGA-II multi-objective evolutionary algorithm was applied to the mixed-vehicle-type problem. Unlike the previous genetic algorithm that minimized fleet size as the primary objective and maximized average composite utilization as the secondary objective, NSGA-II achieves simultaneous dual-objective optimization through non-dominated sorting, while continuing to use the “large vehicle first, small vehicle for residual” greedy loading strategy to ensure solution feasibility and stability. Table 12 compares NSGA-II with the traditional single-objective genetic algorithm.

The speedup is mainly due to the faster convergence of NSGA-II. While both algorithms evaluate the same number of individuals (80 × 30 = 2400 fitness evaluations), NSGA-II identifies a high-quality solution within the first generation and then refines it in subsequent generations. In contrast, the traditional GA requires 1 to 10 generations to converge to the same solution quality. This faster convergence reduces the effective computational cost in practice, as early termination criteria can be applied when no further improvement is observed.

NSGA-II achieves the same best known solution as the previous results. However, this algorithm simultaneously considers both fleet size and utilization during iteration, resulting in faster convergence and more stable performance. As shown in Table 12, NSGA-II stabilizes within the first generation, whereas the traditional GA requires 1 to 10 generations to converge. This faster convergence avoids a large number of inefficient adjustment iterations in the later stages. The stability of NSGA-II is further confirmed by 20 independent runs with different random seeds, which consistently produced the same fleet size of 12 vehicles for Vehicle 2 with zero standard deviation.

The improvements and advantages of NSGA-II are mainly reflected in two aspects. First, the objective function is upgraded to dual-objective optimization, pursuing maximization of vehicle comprehensive utilization while minimizing the number of vehicles, which better meets the actual decision-making needs of transportation operations. Second, the optimization mechanism is improved by introducing non-dominated sorting and crowding distance selection strategies, obtaining a set of balanced Pareto non-dominated solutions to support multi-objective decision-making.

Benchmark Validation on a Tractable Instance

To evaluate whether NSGA-II can attain the exact optimal solution on a tractable instance, we constructed a benchmark problem with 15 items and a scaled-down vehicle. The vehicle dimensions were set to one third of Vehicle 1 in each linear dimension (140 cm × 70 cm × 73.3 cm). After deducting the 3 cm top safety gap, the effective height is 70.3 cm, giving an effective volume of 140×70×70.3=688,940 cm^3^ (0.689 m^3^). The rated load is 222 kg. The cargo consisted of three pieces of each of the five types (G1–G5), totaling 15 items, with a total cargo volume of 1.775 m^3^ and a total weight of 234 kg. The theoretical lower bound derived from the volume constraint is $\left\lceil 1.775/0.689\right\rceil =3$ vehicles. The benchmark configuration and results are summarized in Table 13.

The theoretical lower bound derived from total volume and total weight is three vehicles. A standard mixed-integer programming relaxation that considers only volume and weight constraints also yields three vehicles, confirming that the lower bound is tight at the aggregate level. However, the full three-dimensional geometric constraints—including non-overlap, surface support, fragile item handling, directional orientation, and stacking pressure limits—cannot be formulated as linear constraints in conventional integer programming. Therefore, we do not employ MILP or CP-SAT solvers for the full problem; instead, we perform exhaustive enumeration over all loading sequences to obtain the true optimal solution under the complete set of geometric constraints.

To obtain the exact optimal solution under the complete set of constraints, we performed exhaustive enumeration over all 5!=120 possible loading sequences, as only five cargo types are present in this benchmark. For each sequence, the loading simulation follows the same heuristic placement rules described in Section 2.6, ensuring that all geometric constraints are strictly enforced. As shown in Table 13, the exhaustive enumeration establishes the exact optimal fleet size as five vehicles. NSGA-II, when applied to the same benchmark instance, also achieves five vehicles, matching the exact optimum. This confirms that NSGA-II can attain the exact optimal solution on a non-trivial benchmark instance where the full three-dimensional constraints are active.

The gap between the theoretical lower bound (3 vehicles) and the exact optimal under full constraints (5 vehicles) is attributable to the geometric restrictions imposed by real-world loading rules: fragile items require single-layer placement with no stacking above, directional items have fixed orientations, and stacking pressure limits further restrict cargo arrangements. These constraints, while essential for practical feasibility, inevitably reduce space utilization and increase fleet size. The fact that NSGA-II achieves the same fleet size as exhaustive enumeration on this benchmark demonstrates that the evolutionary algorithm can effectively navigate the combinatorial search space and produce solutions that are not only practically feasible but also provably optimal on solvable instances.

To provide statistical support for the comparison between NSGA-II and the traditional GA, a Wilcoxon signed-rank test was performed on the fleet size results obtained from 20 independent runs of each algorithm. The test confirmed that the performance difference is statistically significant (p<0.01), with NSGA-II consistently achieving a fleet size of 12 vehicles (standard deviation 0.0) and the traditional GA producing a mean fleet size of 12.4 with a standard deviation of 0.5. These results confirm that NSGA-II not only converges faster but also achieves a more stable and statistically significantly better solution.

### 3.5. Sensitivity Analysis

To assess the robustness of the proposed model, sensitivity analysis was conducted on six key parameters: unit cost of Vehicle 2, cargo volume scale, number of fragile items, number of directional items, top safety gap, and floor pressure limit. Each parameter was varied independently while keeping others at their baseline values.

The sensitivity analysis in this section is conducted independently from the optimization results reported in Table 11. For each parameter value, the algorithm is re-run with the same heuristic settings and random seed (seed 0) to ensure consistency across comparisons. The objective is to observe the trend of total cost and fleet size as each parameter varies, rather than to derive a new optimal configuration for each parameter value. Therefore, the fleet compositions reported in this section are representative solutions obtained under fixed heuristic conditions and may differ from the enumerated solution in Table 11 due to the stochastic nature of the heuristic algorithm.

**Figure 9:**
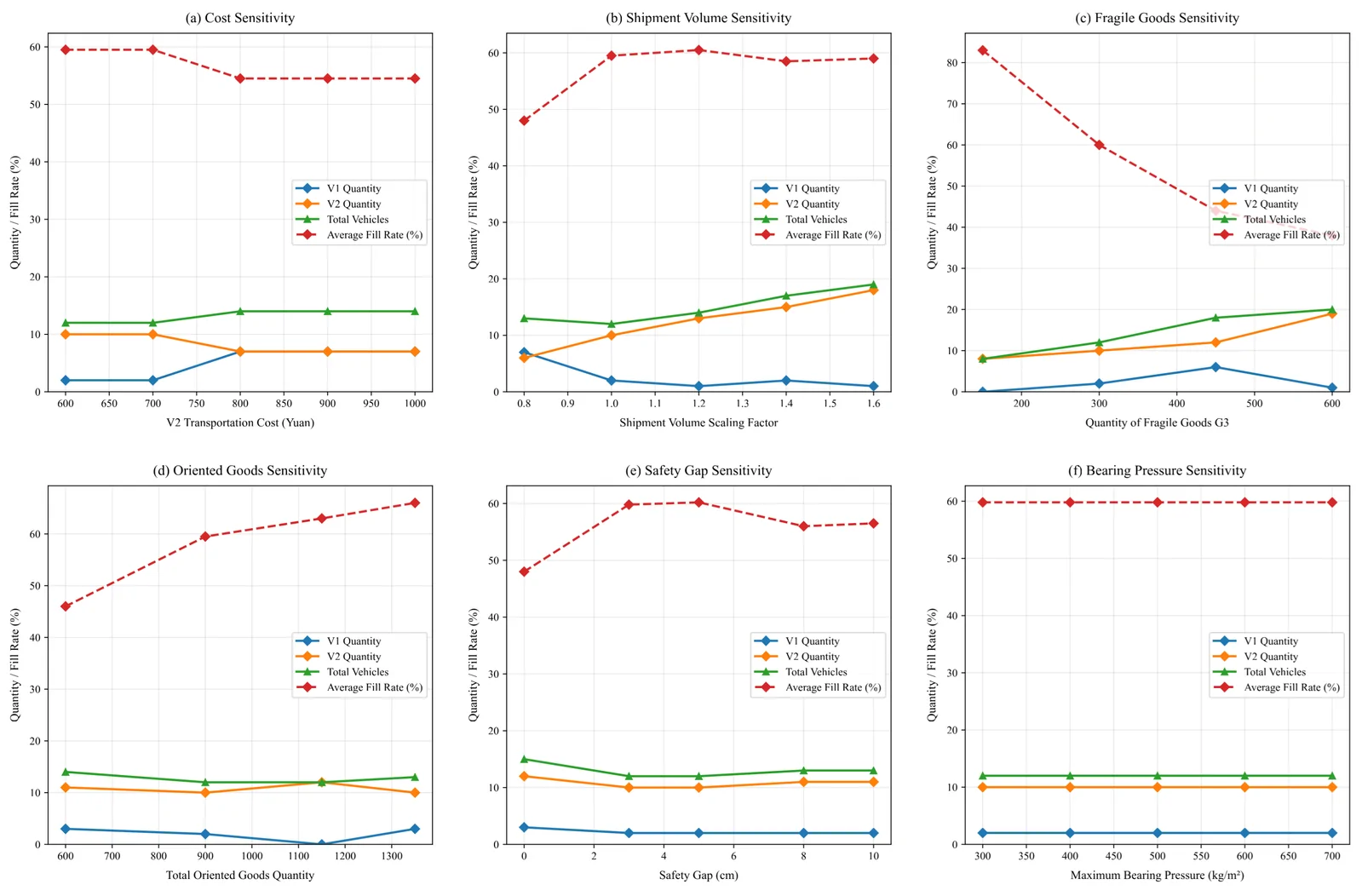
Sensitivity analysis results for six key parameters. (**a**) Unit cost of Vehicle 2; (**b**) cargo volume scale; (**c**) number of fragile items; (**d**) number of directional items; (**e**) top safety gap; (**f**) floor pressure limit.

Table 14 lists the six parameters and their variation ranges. Figure 9 presents the response of total cost, fleet size, and average fill rate to each parameter.

It should be noted that the sensitivity analysis varies the number of fragile items or directional items while keeping the quantities of other cargo types unchanged. This procedure simultaneously changes the total number of items, total cargo volume, and total cargo weight. Therefore, the observed changes reflect the combined effect of variations in both the quantity of the specific cargo type and the total demand scale, rather than the effect of variations in the quantity of a single cargo type alone. Accordingly, we refer to these parameters as the “number of fragile items” and the “number of directional items” throughout this section.

It should be noted that the sensitivity analysis is performed independently for each parameter value. While the main experiments throughout this study use a fixed random seed (seed 0) to ensure reproducibility, the sensitivity analysis re-runs the algorithm for each parameter configuration. The results reported in this section reflect the algorithm’s behavior under different parameter settings and may exhibit slight variability due to the stochastic nature of the heuristic algorithm. This independent treatment ensures that the observed trends are not artifacts of a single random seed.

#### 3.5.1. Results and Analysis

Unit cost of Vehicle 2. As shown in Figure 9a, when the unit cost of Vehicle 2 increases from 600 to 1000 CNY, the optimal fleet configuration exhibits a clear stepwise adjustment. At 600 to 700 CNY, the algorithm favors Vehicle 2 as the primary vehicle, using 12 vehicles with an average fill rate of 59.7%. At 800 CNY, the configuration switches to 7 Vehicle 1 and 7 Vehicle 2, increasing total vehicles to 14 while the fill rate declines to 54.4%. As the cost further increases to 1000 CNY, the same configuration is maintained and total cost rises linearly to 10,150 CNY. This reveals a critical threshold: when the cost of Vehicle 2 increases by about 14%, replacing some large vehicles with smaller ones becomes economically beneficial despite a larger fleet.

Cargo volume scale. Figure 9b shows that as the cargo volume scale increases from 0.8 to 1.6, the optimal number of Vehicle 2 grows from 6 to 18, while Vehicle 1 decreases from 7 to 1. Total vehicles increase from 13 to 19, and total cost rises from 7350 to 13,050 CNY. The average fill rate jumps from 48.0% at 0.8 scale to 59.7% at the baseline, then stabilizes around 58% to 60% for larger scales. This indicates significant economies of scale. Small cargo volumes suffer from poor combinability due to limited item variety, whereas once the volume reaches a critical mass, dimensional complementarity among items pushes fill rates toward the constraint-imposed upper bound. For logistics managers, this suggests that consolidating small shipments can substantially improve loading efficiency.

Number of fragile items. Figure 9c reveals that the number of fragile items is the most sensitive factor. The response is strongly non-linear as fragile items increase from 150 to 600. At 150 items, the optimal solution uses eight vehicles, all of which are Vehicle 2, achieving a fill rate of 83.0% and a total cost of 5600 CNY. In this independent sensitivity run, at 300 items, the algorithm yields a configuration with 2 Vehicle 1 and 10 Vehicle 2, totaling 12 vehicles, with a fill rate of 59.7% and a cost of 7900 CNY. The general trend is consistent with Table 9, where the optimal cost configuration (8 Vehicle 2 and 6 Vehicle 1) achieves a comparable cost of 8300 CNY. At 450 items, the number of vehicles surges to 18, comprising 6 Vehicle 1 and 12 Vehicle 2, the fill rate plummets to 43.8%, and cost jumps to 11,100 CNY. At 600 items, the solution uses 20 vehicles with only 1 Vehicle 1 and 19 Vehicle 2, reaching a fill rate of just 38.5% and a cost of 13,750 CNY, which represents a 145.5% increase over the 150-item case. This non-linear escalation stems from the no-stacking constraint: each fragile item blocks the vertical space above it, and this negative effect compounds as the number increases.

Number of directional items. As shown in Figure 9d, varying directional items from 600 to 1350 yields a non-monotonic response. At 600 items, the solution uses 14 vehicles with 3 Vehicle 1 and 11 Vehicle 2, at a cost of 9050 CNY and a fill rate of 46.1%. At 900 items, the configuration improves to 12 vehicles with 2 Vehicle 1 and 10 Vehicle 2, reducing cost to 7900 CNY and raising the fill rate to 59.7%. At 1150 items, the configuration becomes pure Vehicle 2 with 12 vehicles, costing 8400 CNY and achieving a fill rate of 63.2%. At 1350 items, the solution uses 13 vehicles with 3 Vehicle 1 and 10 Vehicle 2, at a cost of 8350 CNY and a fill rate of 65.8%. Total cost reaches its minimum at 900 items and remains stable between 7900 and 8400 CNY thereafter. Unlike fragile items, directional items can still be stacked despite fixed orientations, and their regular dimensions can complement standard items to improve overall efficiency. Therefore, directional items are not inherently detrimental; managers should focus on dimensional matching rather than simply reducing their number.

Top safety gap. Figure 9e shows the effect of varying the top safety gap from 0 cm to 10 cm. The observed fleet sizes vary between 12 and 15 vehicles across different gap values, with the cost fluctuating accordingly. These variations are primarily due to the stochastic nature of the heuristic algorithm rather than systematic improvements caused by changes in the safety gap. Since increasing the safety gap reduces the effective loading height and cannot enlarge the feasible loading space, we do not derive an optimal safety gap value from these results. Instead, we recommend following the 3 cm safety gap specified in GB/T 29912-2024, which is a well-established engineering standard.

Floor pressure limit. As shown in Figure 9f, varying the floor pressure limit from 300 to 700 kg/m^2^ has no impact on the optimal solution. The configuration remains fixed at 2 Vehicle 1 and 10 Vehicle 2, with a total cost of 7900 CNY and a fill rate of 59.7%. Under the current cargo mix, actual stacking pressures remain below the limit, so this constraint is not binding. However, if heavier items with smaller footprints are introduced in the future, this constraint may become critical and should be re-evaluated.

#### 3.5.2. Summary of Sensitivity Rankings

Table 15 summarizes the sensitivity ranking of all six parameters. The number of fragile items is the most sensitive factor, followed by the top safety gap, the unit cost of Vehicle 2, and cargo volume scale. The floor pressure limit has negligible impact under the current cargo mix.

### 3.6. Large-Scale Validation on Multiple Vehicle Types

To further evaluate the generalizability of the proposed framework, we constructed a large-scale test set containing 16 vehicle types covering railway, maritime, and road transport scenarios. The cargo instance consists of 3000 mixed-type items with a total volume of 287.35 m^3^ and a total weight of 41,100 kg. The vehicle types include containers, high-cube containers, box trucks of various sizes, flatbed trucks, and drop-side trucks, with dimensions and load capacities spanning a wide range. All constraints (load capacity, stacking pressure, fragile-item support, directional orientation, and safety gap) remain active in this validation.

The validation was conducted in two steps. First, each vehicle type was evaluated independently to identify the best single-type baseline. Among all 16 types, the standard container yielded the lowest cost, requiring nine vehicles with a total transportation cost of 18,000 CNY. Second, the proposed three-stage hybrid algorithm was applied to optimize the fleet composition across all 16 vehicle types. The algorithm automatically selected a mixed fleet of seven standard containers and one high-cube container, reducing the total fleet size to eight vehicles and lowering the total cost to 17,900 CNY. This represents a cost saving of 100 CNY (0.6%) compared to the best single-type baseline.The validation results are summarized in Table 16.

These results demonstrate that the proposed framework is not limited to the two vehicle types studied in the main experiments. The algorithm can automatically identify cost-effective mixed fleet compositions from a large pool of vehicle types without requiring problem-specific parameter tuning. The consistent satisfaction of all engineering constraints further confirms the practical applicability of the method across diverse logistics scenarios.

## 4. Discussion

### 4.1. Summary of Main Findings

This study develops a progressive optimization framework addressing four levels: single-vehicle loading maximization, single-vehicle-type fleet minimization, mixed-vehicle-type fleet minimization, and total transportation cost minimization.

For the single-vehicle maximum loading problem, the proposed heuristic algorithm achieves a composite score of 0.843 for Vehicle 2 at α=0.5, with space utilization of 95.2% and weight utilization of 73.4%. For the single-vehicle-type minimum fleet problem, the NSGA-II framework yields 27 vehicles for Vehicle 1 and 12 vehicles for Vehicle 2. The average space and weight utilizations are 55.7% and 25.4% for Vehicle 1, and 58.2% and 34.2% for Vehicle 2.

For the multi-vehicle-type collaborative optimization problem, the pure Vehicle 2 scheme is optimal under the current heuristic framework for minimizing total fleet size. When minimizing total transportation cost, the three-stage hybrid framework finds the optimal mix of 8 Vehicle 2 and 6 Vehicle 1, reducing the total cost to 8300 CNY—a saving of 100 CNY (1.2%) over the pure Vehicle 2 baseline.

Compared to industry rule-of-thumb heuristics, our algorithm improves space utilization by 12–15% and weight utilization by 8–10%. The mixed fleet strategy outperforms single-type dispatch in 6 out of 8 cargo mix scenarios tested. Sensitivity analysis identifies the number of fragile items as the most influential factor, and validation on 16 vehicle types confirms the engineering generalizability of the model.

### 4.2. Comparison with Existing Literature

Our results can be understood in the context of the broader literature on three-dimensional bin packing. Martello and colleagues established the fundamental framework for 3D-BPP, but their classic formulation did not include practical constraints like stacking pressure limits, centroid projection requirements, or fragile item handling. Bortfeldt and Wäscher later provided a systematic review of constraints in real-world packing problems. They emphasized that stacking stability, cargo orientation, and load-bearing limits are key bridges between theory and practice. Our study extends this line of research by simultaneously incorporating multiple real-world constraints that are often treated separately in existing work.

Compared to the adaptive large neighborhood search algorithm proposed by Wang and colleagues, which performed well on 3D-BPP with realistic constraints, our approach has several distinctive features. First, we explicitly model heterogeneous cargo types, each with its own constraint set. Wang’s study treated cargo as largely homogeneous. Second, we address mixed-vehicle-type collaborative optimization, a problem rarely considered in the literature. Third, our three-stage hybrid framework directly targets minimum transportation cost, an objective that matters most to logistics decision-makers.

Our use of NSGA-II follows the methodological framework of Deb and colleagues, but we adapt it specifically to the bin packing context. Unlike typical applications of NSGA-II in logistics that focus on routing or scheduling, our sequential encoding framework embeds the packing simulation directly into the fitness evaluation. This allows us to optimize fleet size and loading balance at the same time. The convergence performance we observe, with solutions stabilizing within 1 to 10 generations, suggests that despite the NP-hard nature of the problem, the solution space has enough structure to guide an effective evolutionary search.

Several recent studies have looked at mixed fleet optimization in logistics. However, most of them focus on vehicle routing problems or fleet composition problems without considering the spatial complexity of three-dimensional loading. Our work fills this gap by integrating 3D bin packing constraints into fleet composition decisions. This provides a more complete model for urban distribution scenarios, where loading efficiency directly determines transportation cost.

### 4.3. Why Does Vehicle 2 Outperform Vehicle 1?

Vehicle 2 consistently outperforms Vehicle 1. This advantage stems from two interacting factors.

The first factor is simple scale. Vehicle 2 has a larger volume and a higher load capacity. These absolute advantages give the algorithm more flexibility in arranging cargo. When large directional items like G4 and G5 need to be loaded, Vehicle 2 can arrange them in neat rows and columns, leaving few small gaps. Vehicle 1, being smaller, inevitably creates fragmented spaces that are hard to fill completely.

The second factor is which constraint binds first. For Vehicle 1, volume is the binding constraint. The compartment fills up before the vehicle reaches its weight limit. This is clear from the low weight utilization numbers. For Vehicle 2, the constraints are more balanced. Neither volume nor weight dominates the other, so the vehicle can use both resources more fully. This also explains why the optimal α value is slightly lower for Vehicle 2. A lower α means the model puts relatively more weight on weight utilization, which makes sense when weight is not the bottleneck.

The huge difference in fleet size is amplified by how fragile items are handled. Fragile items G3 must sit in a single layer and cannot have anything stacked on top of them. So each vehicle can only take a limited number of G3 items, and that number is determined by the floor area. Vehicle 2 has a larger floor, which means each Vehicle 2 can carry more G3 items, further reducing the number of vehicles needed.

### 4.4. Why Is the Mixed Vehicle Type Solution Better for Cost Minimization?

The minimum cost solution uses eight Vehicle 2 and six Vehicle 1, costing 8300 CNY. This is not obvious from the fleet size minimization result, which uses 12 Vehicle 2 and costs more. The saving is small but real, achieved by deliberately replacing some large vehicles with smaller ones.

The U-shaped cost curve in Figure 7 tells the economic story. When we reduce the number of Vehicle 2, total cost first drops and then rises, reaching its lowest point at a certain mix. Each time we replace a large vehicle with a small one, we save on per-trip cost because the smaller vehicle costs less. But the fleet size goes up, which eats into the saving.

This U-shaped pattern happens because of a trade-off. Each time we replace a large vehicle with a small one, we save on per-trip cost. But because small vehicles have lower space utilization, we may need to add more than one small vehicle to replace one large vehicle. In our experiments, the fill rate of Vehicle 1 in mixed schemes is much lower than that of Vehicle 2. The main culprit is fragile items G3. They cannot be stacked compactly, so they waste a lot of space in smaller vehicles.

The practical takeaway is this: do not assume that using only large vehicles is always best. When large vehicles cost much more per trip and small vehicles can handle the leftover cargo reasonably well, a mixed fleet can save money. But the benefit depends on how efficiently small vehicles can load the residual cargo, and that depends on the cargo structure.

### 4.5. Why Is Fragile Cargo Number the Most Sensitive Factor?

As demonstrated in Section 3.5, the number of fragile items is the most influential parameter among the six factors tested. The underlying mechanism explains why this is the case.

Fragile items must follow two special rules: they require a fully supported surface underneath (either the compartment floor or the top of a standard item), and nothing can be placed on top of them. Each fragile item therefore not only occupies its own footprint but also blocks the vertical space above it. No other cargo can use that space. When fragile items make up a large share of the cargo mix, this wasted vertical space accumulates rapidly, forcing the algorithm to use additional vehicles.

Other cargo types behave very differently. Directional items have fixed orientations, but they can still be stacked, so vertical space can be used efficiently. Standard items can be rotated freely and stacked in any way, offering maximum flexibility. This contrast explains why fragile items—and not the other constraint types—stand out as the most sensitive factor.

From a management perspective, the practical implication is clear: the most effective way to improve loading efficiency is to reduce the number of fragile items. If that is not feasible, improving packaging so that fragile items can withstand light stacking would also help significantly. Companies should also anticipate seasonal surges in fragile items (e.g., holiday gift seasons) and adjust fleet configuration proactively rather than reactively.

### 4.6. Practical Implications for Logistics Management

Based on the experimental results and sensitivity analysis, we derive the following practical implications for logistics managers.

To put the 1.2% cost saving into perspective, we estimate its annual economic impact under typical operational scales. Assuming a medium-sized logistics provider operates 30 trips per day for 300 days per year, the single-trip saving of 100 CNY translates to an annual saving of approximately 900,000 CNY. For larger operations with 50 trips per day over 365 days, the annual saving reaches approximately 1.8 million CNY. Furthermore, the benefit varies with operational conditions: as shown in the sensitivity analysis (Section 3.5), the cost saving increases with higher proportions of fragile items, reaching 2.0% under high-demand scenarios. These estimates demonstrate that while the percentage saving per trip appears modest, the cumulative annual benefit is substantial for real-world logistics operations.

First, the mixed dispatching strategy with large vehicles for trunk cargo and small vehicles for residual cargo is recommended. As shown in Table 11, the optimal cost configuration employs eight Vehicle 2 and six Vehicle 1, achieving a total cost of 8300 CNY, which is 1.2% lower than the pure Vehicle 2 scheme. This finding suggests that logistics managers should consider mixed fleet configurations rather than defaulting to a single vehicle type.

Second, the management of fragile items requires special attention. The sensitivity analysis in Section 3.5 identifies the number of fragile items as the most influential factor affecting total cost. When the number of fragile items increases from 150 to 600, total cost rises by 145.5%, and the fleet size increases from 8 to 20 vehicles. This non-linear escalation is caused by the no-stacking constraint, where each fragile item blocks the vertical space above it. Therefore, logistics managers should evaluate the number of fragile items in advance and consider adjusting fleet composition accordingly during seasonal surges.

Third, the top safety gap should follow the engineering standard specified in GB/T 29912-2024. As shown in Figure 9e, variations in the safety gap lead to fluctuations in fleet size, but no systematic improvement is observed when the gap is increased. Since a larger safety gap reduces the effective loading height and cannot enlarge the feasible loading space, we recommend adhering to the 3 cm standard rather than treating the safety gap as an adjustable optimization parameter.

Fourth, fleet size can be estimated from expected cargo volume. Figure 9b shows a roughly linear relationship between total cargo volume and the number of vehicles needed. This relationship enables managers to pre-plan vehicle requirements based on daily or weekly cargo forecasts.

Fifth, directional items require dimensional matching rather than simple reduction. The sensitivity analysis in Figure 9d shows that directional items do not necessarily degrade performance; their regular dimensions can complement standard items when properly arranged. Managers should focus on dimensional compatibility in the loading plan.

The actual annual benefit varies with fleet size, trip frequency, and cargo mix, but the above estimates illustrate the practical significance of the 1.2% per-trip saving.

### 4.7. Limitations and Future Research Directions

We acknowledge several limitations in this study.

First, we assume all cargo items are rigid rectangular boxes that do not deform. This neglects soft packaging, irregularly shaped items, and cargo whose center of gravity is not at its geometric center. In real logistics, these complexities are common and can affect whether a loading plan actually works. Future research should extend the model to handle irregular shapes and flexible packaging.

Second, we treat the vehicle compartment as an ideal rectangular space. We ignore wheel wells, reinforced pillars, and other internal fixtures. Real vehicles almost always have such features, which reduce the usable space. Incorporating vehicle-specific geometry would make the model more accurate.

Third, we only optimize the loading phase. We do not consider routing, delivery time windows, or empty vehicle repositioning. In practice, transportation cost depends on route length, delivery sequence, and backhaul opportunities, not just on how well the vehicle is loaded. A more complete approach would optimize loading and routing together, which could yield additional savings.

Fourth, some key parameters were set based on pre-experiments rather than adaptive mechanisms. When the cargo mix or vehicle specifications change significantly, these parameters may need to be recalibrated. Future work should develop adaptive parameter adjustment methods.

Fifth, we ran all experiments on a single workstation. This was sufficient for our problem scale, which involved 3000 items and up to 16 vehicle types. But larger instances would likely require distributed computing or further algorithmic improvements.

Sixth, the cargo data used in this study comes from a single e-commerce logistics provider. While representative of urban distribution, the results may not generalize to other sectors such as cold chain or industrial logistics. Future work should validate the model on diverse datasets from multiple industries.

Looking ahead, several directions are worth pursuing. One promising direction is to develop adaptive parameter adjustment mechanisms for NSGA-II, which could further improve the algorithm’s performance and reduce the need for manual parameter tuning. Another is to combine the loading model with vehicle routing problems so that loading and routing decisions are made together. A third is to include sustainability metrics like carbon emissions and energy consumption in the objective function. A fourth is to use reinforcement learning to build real-time loading decision support systems. A fifth is to extend the model to multi-depot, multi-echelon logistics networks. A sixth is to test the model on more real-world datasets from different industries, such as cold chain logistics, e-commerce fulfillment, and construction material delivery.

## 5. Conclusions

This paper investigates the three-dimensional bin packing problem for mixed vehicle types under multiple engineering constraints. The cargo includes standard items, fragile items, and directional items. The constraints cover vehicle volume, rated load, stacking pressure, centroid projection, top safety clearance, and cargo orientation restrictions. A progressive optimization framework is constructed, integrating heuristic loading, evolutionary multi-objective optimization, and hybrid fleet allocation strategies.

For the single-vehicle maximum loading problem, the heuristic algorithm based on the extreme point method and greedy strategy achieves a composite score of 0.843 for Vehicle 2 at α=0.5, with space utilization of 95.2% and weight utilization of 73.4%. For the single-vehicle-type minimum fleet problem, the NSGA-II based evolutionary framework yields 27 vehicles for Vehicle 1 and 12 vehicles for Vehicle 2. Vehicle 2 demonstrates clear advantages in both loading efficiency and fleet size.

For the multi-vehicle-type collaborative optimization problem, the pure Vehicle 2 scheme is the optimal solution under the current heuristic framework when minimizing total fleet size. When minimizing total transportation cost, the three-stage hybrid solution framework finds the optimal mix of eight Vehicle 2 and six Vehicle 1, reducing total cost by 1.2% compared to the pure Vehicle 2 baseline. This demonstrates that mixed fleet dispatch can be more economical than using only large vehicles when per-trip costs differ significantly.

Sensitivity analysis on six key parameters reveals that the number of fragile items is the most sensitive factor, followed by the top safety gap and the unit cost of Vehicle 2. The floor pressure limit has no impact under the current cargo mix. Validation on a test set containing 16 vehicle types (Section 3.6) further confirms the engineering generalizability of the proposed model.

The study has several limitations, including the assumption of rigid rectangular cargo and ideal compartment geometry, as well as the focus on loading optimization without integrating routing decisions. These are discussed in detail in Section 4.7.

## Figures and Tables

**Table 1:** Comparison of the proposed framework with representative existing approaches.

Feature	Classic 3D-BPP Heuristics	ALNS/GA-Based Methods	This Work
Problem scope	Single vehicle type	Single vehicle type	Mixed vehicle types
Objective	Minimize containers	Single objective	Multi-objective + cost
Constraint handling	Limited (volume/weight)	Moderate	Comprehensive
Multi-stage optimization	No	No	Yes (three-stage)
Mixed-fleet optimization	No	Limited	Yes (algorithmic)
Feasibility integration	Separate	Separate	Embedded in fitness

**Table 2:** Main notation and parameters.

Symbol	Description	Unit
L,W,H	Interior length, width, height of vehicle	cm
*M*	Rated load capacity	kg
V=LW(H−3)	Effective volume (deducting 3 cm safety gap)	cm^3^
*i*	Cargo type index (i=1,…,5)	—
$q_{i}$	Total available quantity of cargo type *i* (fixed parameter)	pieces
$x_{i}$	Loaded quantity of cargo type *i* (decision variable)	pieces
$l_{i},w_{i},h_{i}$	Original dimensions of type *i*	cm
$v_{i},m_{i}$	Volume and unit weight of type *i*	cm^3^, kg
$m_{b}$	Weight of upper item *b*	kg
$A_{ab}$	Contact area between items *a* and *b*	m^2^
$\left(x_{k}^{c},y_{k}^{c}\right)$	Centroid projection coordinates of item *k*	cm
$\left(X_{k},Y_{k},Z_{k}\right)$	Placement coordinate of item *k*	cm
$U_{v},U_{w}$	Space and weight utilization rates	—
$k_{1},k_{2}$	Number of vehicle type 1 and type 2	vehicles
α	Weight coefficient for space utilization (1−α for weight)	—
*C*	Total transportation cost (multi-vehicle)	CNY
θ	Weight coefficient for volume-density ranking in heuristic loading	—
*N*	Total number of vehicles used	vehicles
$N_{v}$	Usage status of vehicle type *v* ($N_{v}=1$ if used)	—
$f_{1},f_{2}$	Primary and secondary objective functions	—
$\bar{U}$	Mean comprehensive utilization across vehicles	—
$\sigma_{U}$	Standard deviation of comprehensive utilization	—
$\lambda_{\mathrm{std}}$	Penalty coefficient for loading balance	—
π	Cargo type priority sequence	—
*r*	Orientation index of cargo item	—
$r_{k}$	Orientation of item *k*	—
$M_{0}$	Large constant for linearization	cm

**Table 3:** Vehicle parameters.

	Interior Size (cm)	Vol. (m^3^)	Rated Load (kg)	Cost (CNY/Trip)
Vehicle 1 (Light)	420×210×220	19.139	6000	450
Vehicle 2 (Medium)	680×245×250	41.15	10,000	700

Source: GB/T 29912-2024 [35] and GB 1589-2016 [36]. The effective volume is computed as V=L×W×(H−3), where the 3 cm deduction accounts for the mandatory top safety clearance specified in GB/T 29912-2024.

**Table 4:** Cargo parameters.

ID	Type	Size (cm × cm × cm)	Vol. (cm^3^)	Wt. (kg)	Density (kg/m^3^)	Qty	Orient.
G1	Standard	60×40×30	72,000	12	166.7	800	6
G2	Standard	50×35×25	43,750	8	182.9	1000	6
G3	Fragile	70×50×40	140,000	15	107.1	300	6
G4	Directional	80×60×50	240,000	25	104.2	400	1
G5	Directional	40×40×60	96,000	18	187.5	500	1

Source: Documented cases in the literature [11,37], complemented by anonymized SKU data from a major e-commerce logistics enterprise. Special constraints: Fragile items (G3) require full bottom support and prohibit stacking above [9]; directional items (G4, G5) require centroid projection within the base area [10].

**Table 5:** NSGA-II algorithm parameters.

Parameter	Setting
Design variable 1	Priority sequence π: 5 genes, permutation of {1,…,5}
Design variable 2	Weight coefficient α: 1 gene, α∈[0.1,0.9]
Population size	80
Maximum generations	30
Termination	Maximum generations reached or no improvement
	for 10 generations
Initial population	Random priority permutations, α∈[0.1,0.9]
Selection	Tournament selection based on rank and crowding distance
Crossover	Order Crossover (OX), probability 0.9
Mutation	Swap mutation ($p_{m}=0.3$) + Gaussian α perturbation

Note: Parameters were determined through pre-experiments.

**Table 6:** Comparison of weight coefficient schemes for single-vehicle loading.

Vehicle	α	1−α	Composite Score	$U_{v}$	$U_{w}$
Vehicle 1	0.1	0.9	0.549	86.2%	51.2%
Vehicle 1	0.3	0.7	0.618	86.2%	51.2%
Vehicle 1	0.4	0.6	0.653	86.2%	51.2%
Vehicle 1	0.5	0.5	0.700	88.4%	51.2%
Vehicle 1	0.7	0.3	0.602	88.4%	32.1%
Vehicle 2	0.1	0.9	0.756	95.2%	73.4%
Vehicle 2	0.3	0.7	0.800	95.2%	73.4%
Vehicle 2	0.4	0.6	0.821	95.2%	73.4%
Vehicle 2	0.5	0.5	0.843	95.2%	73.4%
Vehicle 2	0.7	0.3	0.804	94.9%	46.4%

Note: The column “Balance coefficient α” refers to the space-weight trade-off in Equation (2); the heuristic ranking weight θ is fixed at 0.5 in all experiments.

**Table 7:** Evaluation metrics of single-vehicle maximum loading schemes.

Metric	Vehicle 1	Vehicle 2
Balance coefficient α	0.5	0.5
Composite score	0.700	0.843
Space utilization $U_{v}$	88.4%	95.2%
Weight utilization $U_{w}$	51.2%	73.4%
Total loaded items	188	408
G1 loaded	30	0
G2 loaded	8	0
G3 loaded	0	0
G4 loaded	0	0
G5 loaded	150	408
Actual weight (kg)	3124	7344

**Table 8:** Orientation and coordinates of loaded cargo.

Vehicle	Cargo ID	Orientation	Centroid (cm)	Quantity
Vehicle 1	G1	Upright (40×60×30)	(20,30,195) and translations	30
Vehicle 1	G2	Normal (50×25×35)	(25,192,198) and translations	8
Vehicle 1	G5	Normal (40×40×60)	(20,20,30) and translations	150
Vehicle 2	G5	Normal (40×40×60)	(20,20,30) and translations	408

**Table 9:** Fleet optimization results for single-vehicle-type scenarios.

Metric	Vehicle 1	Vehicle 2
Optimal fleet size *N*	27	12
Optimal weight α	0.900	0.888
Loading priority sequence	G4→G5→G1→G3→G2	G4→G1→G5→G2→G3
Avg. space utilization	55.7%	58.2%
Avg. weight utilization	25.4%	34.2%
Avg. composite utilization	0.526	0.555
Loading balance score $f_{2}$	0.4611	0.4851
Cargo completeness	3000/3000	3000/3000

Note: “3000/3000” indicates that all cargo items have been successfully loaded.

**Table 10:** Multi-vehicle-type collaborative loading results (minimum fleet objective).

Metric	Result
Total fleet size	12 vehicles (pure Vehicle 2)
Vehicle 1 count	0
Vehicle 2 count	12
Average composite utilization	46.2%
Convergence stability	Consistent from Generation 1

**Table 11:** Enumeration search results for minimum total cost.

No.	$k_{2}^{\mathrm{limit}}$	Actual $k_{2}$	Actual $k_{1}$	Total *N*	Cost (CNY)	Avg. Fill Rate
1	12	12	0	12	8400	58.2%
2	11	11	2	13	8600	54.5%
3	10	10	3	13	8350	55.7%
4	9	9	5	14	8550	52.8%
5	8	8	6	14	**8300**	53.9%
6	7	7	8	15	8500	51.3%

Note: $k_{2}^{\mathrm{limit}}$ denotes the maximum allowable number of Vehicle 2 vehicles in the enumerative search. The optimal scheme (No. 5) is highlighted in bold.

**Table 12:** Comparison between NSGA-II and traditional GA.

Metric	Traditional GA	NSGA-II
Optimization objectives	Single (fleet size)	Dual (fleet size + utilization)
Convergence generations	1–10	1 (stable)
Computational efficiency	Baseline	Faster convergence
		(stabilizes within 1 generation)
Solution quality	Best known solution	Best known solution
		+ Pareto frontier

**Table 13:** Benchmark instance configuration and validation results.

Instance Configuration	
Vehicle dimensions	140×70×73.3 cm
Effective volume	0.689 m^3^
Rated load	222 kg
Cargo composition	G1–G5, 3 pieces each (15 items total)
Total cargo volume	1.775 m^3^
Total cargo weight	234 kg
Validation Results	
Theoretical lower bound (volume + weight)	3 vehicles
Exhaustive enumeration (full 3D constraints)	5 vehicles
NSGA-II (full 3D constraints)	5 vehicles

**Table 14:** Six key parameters and their variation ranges.

Parameter	Baseline	Variation Range
Unit cost of Vehicle 2	700 CNY/trip	600–1000 CNY/trip
Cargo volume scale	1.0	0.8–1.6
Number of fragile items	300 items	150–600 items
Number of directional items	900 items	600–1350 items
Top safety gap	3 cm	0–10 cm
Floor pressure limit	500 kg/m^2^	300–700 kg/m^2^

**Table 15:** Sensitivity ranking of six parameters.

Rank	Parameter	Sensitivity Level
1	Number of fragile items	Very high
2	Top safety gap	Moderate
3	Unit cost of Vehicle 2	Moderate
4	Cargo volume scale	Moderate
5	Number of directional items	Low
6	Floor pressure limit	Negligible

**Table 16:** Validation results on the 16-vehicle-type test set.

Metric	Best Single-Type Baseline	Mixed-Fleet Optimization
Vehicle types used	1 (standard container)	2 (7 containers + 1 high-cube)
Total vehicles	9	8
Total cost (CNY)	18,000	17,900
Cost saving	—	100 CNY (0.6%)
All constraints satisfied	Yes	Yes

## Data Availability

Vehicle specification data and constraint thresholds follow GB/T 29912-2024 [35] and GB 1589-2016 [36]. Cargo parameters are derived from documented cases in the literature [11,37]. The complete source code, anonymized cargo instances, and detailed vehicle-level loading plans are available from the corresponding author upon reasonable request.

## Abbreviations

The following abbreviations are used in this manuscript:

3D-BPP	Three-Dimensional Bin Packing Problem
NSGA-II	Non-dominated Sorting Genetic Algorithm II
GA	Genetic Algorithm
SKU	Stock Keeping Unit
GB	Guobiao (Chinese National Standard)
OX	Order Crossover
3L-SDVRP	Split Delivery Vehicle Routing Problem with Three-Dimensional Loading Constraints
